# mTOR inhibition suppresses salinomycin-induced ferroptosis in breast cancer stem cells by ironing out mitochondrial dysfunctions

**DOI:** 10.1038/s41419-023-06262-5

**Published:** 2023-11-15

**Authors:** Emma Cosialls, Emeline Pacreau, Clémence Duruel, Sara Ceccacci, Rima Elhage, Christophe Desterke, Kevin Roger, Chiara Guerrera, Romane Ducloux, Sylvie Souquere, Gérard Pierron, Ivan Nemazanyy, Mairead Kelly, Elise Dalmas, Yunhua Chang, Vincent Goffin, Maryam Mehrpour, Ahmed Hamaï

**Affiliations:** 1 Université Paris Cité, INSERM UMR-S1151, CNRS UMR-S8253, Institut Necker Enfants Malades, Team 5 and Ferostem group, F-75015 Paris, France; 2 Ferostem group, F-75015 Paris, France; 3 Proteomic Core Facility, Université de Paris - Structure Fédérative de Recherche - Necker, INSERM US24/CNRS, UAR3633 Paris, France; 4 UFR Médecine-INSERM UMS33, Université Paris-Sud, F94800 Villejuif, France; 5 CNRS, UMR9196, Villejuif, France - Gustave Roussy Cancer Campus, Villejuif, France; 6 Metabolic Core Facility, Université de Paris - Structure Fédérative de Recherche - Necker, INSERM US24/CNRS, UAR3633 Paris, France

**Keywords:** Breast cancer, Cell death

## Abstract

Ferroptosis constitutes a promising therapeutic strategy against cancer by efficiently targeting the highly tumorigenic and treatment-resistant cancer stem cells (CSCs). We previously showed that the lysosomal iron-targeting drug Salinomycin (Sal) was able to eliminate CSCs by triggering ferroptosis. Here, in a well-established breast CSCs model (human mammary epithelial HMLER CD24^low^/CD44^high^), we identified that pharmacological inhibition of the mechanistic target of rapamycin (mTOR), suppresses Sal-induced ferroptosis. Mechanistically, mTOR inhibition modulates iron cellular flux and thereby limits iron-mediated oxidative stress. Furthermore, integration of multi-omics data identified mitochondria as a key target of Sal action, leading to profound functional and structural alteration prevented by mTOR inhibition. On top of that, we found that Sal-induced metabolic plasticity is mainly dependent on the mTOR pathway. Overall, our findings provide experimental evidence for the mechanisms of mTOR as a crucial effector of Sal-induced ferroptosis pointing not only that metabolic reprogramming regulates ferroptosis, but also providing proof-of-concept that careful evaluation of such combination therapy (here mTOR and ferroptosis co-targeting) is required in the development of an effective treatment.

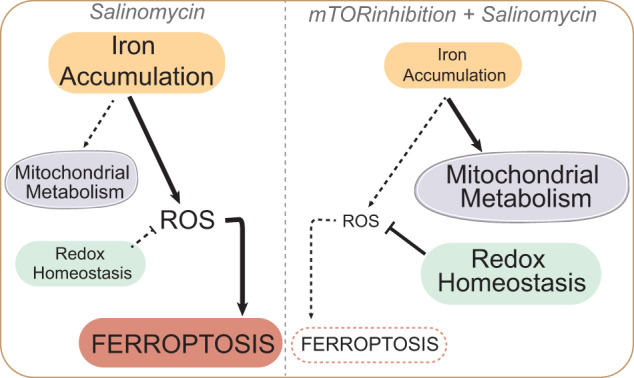

## Introduction

Tumor relapse and metastasis, along with increased resistance to conventional therapies, are a major clinical challenge in curing breast cancer. The therapeutic failure is thought to be caused by a sub-population of highly tumorigenic cells with stem cell properties, termed cancer stem cells (CSCs) [1–3]. We previously showed that breast CSCs are highly sensitive to ferroptosis, a non-apoptotic and iron-dependent cell death while being resistant to conventional therapy compared to bulk tumor cells [4, 5]. Salinomycin (Sal), a polyether antibiotic, selectively kills breast CSCs by ferroptosis in vitro and in vivo. Mechanistically, we have shown in well-established breast CSCs model (human mammary epithelial HMLER CD24^low^/CD44^high^) [6, 7] that Salinomycin triggers ferroptosis by sequestering iron in the lysosomes [4, 8]. However, the specific regulatory mechanisms of Sal-induced ferroptosis are still unexplored.

Recently, accumulating evidence has identified the mTOR pathway, as a critical regulator of ferroptosis [9], sometimes negative [10–12], and other times positive [13, 14]. This oncogenic pathway is one of the most frequently dysregulated pathways in cancer. It is a master controller of cell growth, survival, and metabolism, activated by several factors, including growth factors and nutrients.

The present study confirms the crucial role of mTOR in ferroptosis and indicates that mTOR inhibition suppresses the therapeutic effect of Sal in breast CSCs. Mechanistically, inhibition of mTOR prevents the Sal-induced iron burst and thereby limits iron-mediated oxidative stress. Furthermore, an integrated metabolomics and proteomics approach provides new insights into mitochondria as a key target of Sal action, leading to profound alterations in mitochondrial metabolic pathways prevented by mTOR inhibition. Overall, our work supports that Sal-induced metabolic plasticity is mainly dependent on the mTOR pathway and that its inhibition exerts a protective role against ferroptosis by modulating iron homeostasis, preventing the Sal-induced metabolic burden while decreasing the accumulation of damaged mitochondria. Furthermore, our study underlines that metabolic reprogramming regulates ferroptosis sensitivity, thus opening new opportunities to treat tumors unresponsive to therapies.

## Results

### mTOR inhibition protects cells from Sal-induced cell death

**Fig. 1:**
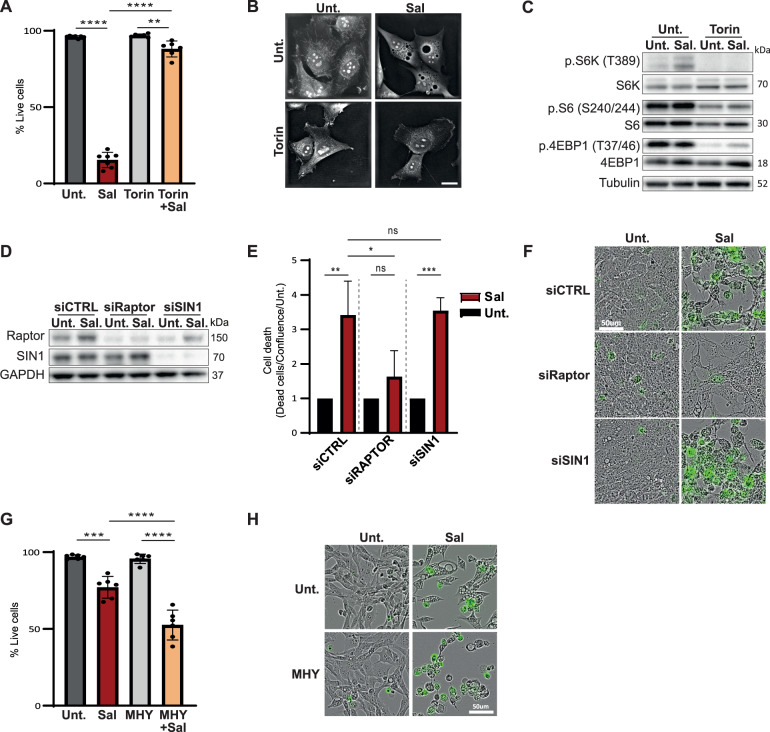
mTOR inhibition prevents cell death induced by Sal. **A**–**C** HMLER CD24L cells were treated with Sal (500 nM), Torin (250 nM), or a combination of both. **A** Cell death was determined by dapi staining coupled with flow cytometry (FC) after 96 h (*n* = 7). **B** Live cell imaging by 3D holotomographic microscopy (Nanolive) after 48 h of treatment. Objective: 63x. Scale bar: 20 μm. **C** Immunoblotting for the indicated mTOR-related protein after 48 h. Tubulin is used as loading control. **D**–**F** HMLER CD24L knockdown for either *RAPTOR* or *SIN1* and then treated as indicated. **D** Immunoblotting for the indicated silenced protein after 48 h. GAPDH is used as loading control. **E**, **F** Acquisition of cell viability using Incucyte Live-cell analysis, (**E**) dead cells were counted by Incucyte® Cytotox Dye Probe over cell confluence normalized on each untreated condition after 120 h of treatment (*n* = 3) and (**F**) Live cell imaging for 96 h of treatment. Objective: 20x. Scale bar: 50 μm. **G**, **H** HMLER CD24L were treated with either Sal (500 nM), MHY1485 (5 μM), or a combination of both for 72 h. **G** cell death was determined by dapi staining coupled with FC (*n* = 7). **H** Live cell imaging as for (**F**). Objective: 20×. Scale bar: 50 μm. Data are presented as: mean ± SD, ANOVA test: *p < 0.05; **p < 0.01; ***p < 0.001; ****p < 0.0001.

To investigate whether mTOR impacts Sal-induced ferroptosis, HMLER CD24^low^ cells were treated with the ATP-competitive mTOR inhibitor Torin [15] in combination with Sal for 96 h. Intriguingly, Torin treatment potently suppressed Sal-induced cell death (Fig. 1A, B, and S1A). Torin blocked the phosphorylation of the mTORC1 substrates: S6, p70S6K and 4EBP1 as expected (Fig. 1C quantifications in S1B). Of note, Sal treatment alone activated the phosphorylation of S6 and S6K (Fig. 1C quantification in S1B). As expected, ferroptotic inhibitors including antioxidant ferrostatin-1, liproxstatin-1, and vitamin E affected Sal-induced cell death (Fig. S1C). More interestingly, Sal induced the expression of PTGS2/COX2 protein, a marker of ferroptotic cell death, from 48 h-treatments, which is inhibited under Torin (Fig. S1D). To confirm the protective effect of mTOR inhibition, other inhibitors were tested: ATP-competitive inhibitors (Torin-2 or AZD8055) also inhibited Sal-induced cell death to the same extent as Torin, while the well-known mTORC1 inhibitor Rapamycin had a weaker effect (Fig. S1E, S1F and S1G). Next, siRNA targeting subunits of mTORC1 (si*RAPTOR*, regulatory-associated protein of mTOR) or mTORC2 (si*SIN1*, mammalian stress-activated protein kinase-interacting protein 1*)* were used (Fig. 1D). Upon Sal treatment, only cells knocked-down for RAPTOR prevented Sal-induced cell death (Fig. 1E, F). These data therefore suggest that the suppression of Sal-induced cell death is driven specifically by mTOR inhibition, mainly through mTORC1. Furthermore, the use of MHY1485, a compound designed to activate mTOR [16], potentiated Sal-induced cell death (Fig. 1G, H). Taken together, these data highlight the crucial role of mTOR signaling in the regulation of Salinomycin-induced cell death.

### mTOR inhibition prevents ROS and iron accumulation

**Fig. 2:**
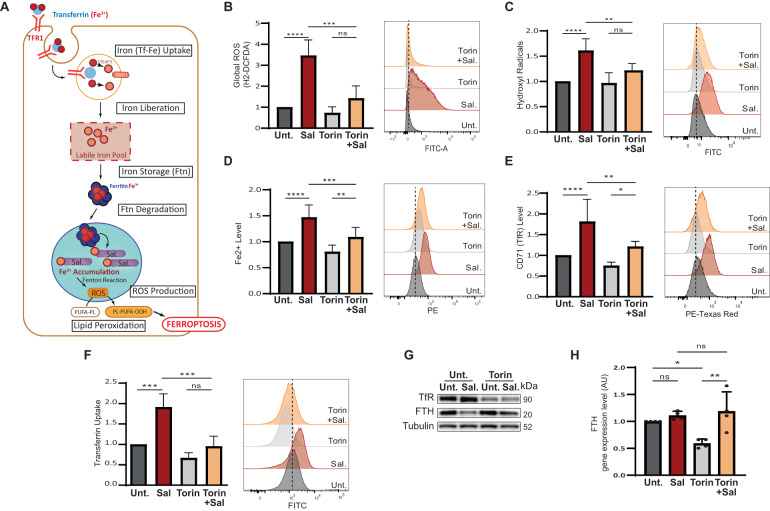
mTOR inhibition reduces pro- ferroptotic hallmarks induced by Sal. **A** Iron entry into the cell is mediated by the binding of Fe^3+^-Tf complex to TFR and its subsequent endocytosis. Iron is released under the acidic environment of the endosome, and is reduced to Fe2+ by STEAP3 and transported into the cytosol by DMT1. Free iron, constituting the labile iron pool, can be used (in mitochondria, …), exported, or stored in ferritin. Low iron availability triggered the degradation of ferritin in mitochondria. Salinomycin sequestered iron in the lysosomes, triggering a cellular iron-depletion response that induced an increase in iron entry, and an increase ferritin degradation, it results in an accumulation of iron in the lysosome and a subsequent massive ROS production (by the Fenton reaction) leading to a considerable lipid peroxidation and ultimately cell death. **B**–**G** HMLER CD24L were treated with Sal, Torin, or a combination of both for 48 h. **B** Global ROS level determined by H2-DCFDA staining coupled with FC (*n* = 4). **C** Hydroxyl Radical level determined by HPF staining coupled with FC (*n* = 6). **D** Fe^2+^ level determined by FerroOrange staining coupled with FC (*n* = 9). **E** TFR level determined by anti-CD71 staining coupled with FC (*n* = 7). **F** Transferrin Uptake was determined by Alexa-488-Transferin staining coupled with FC (*n* = 4). **G** Immunoblotting for the indicated iron-related protein. Tubulin is used as loading control. **H** FTH gene expression level by RT-qPCR. Data are presented as: mean ± SD, ANOVA test: *p < 0.05; **p < 0.01; ***p < 0.001; ****p < 0.0001.

Figure 2A summarizes iron homeostasis and the mechanisms by which Sal leads to iron accumulation, excessive ROS production, and lipid peroxidation, ultimately triggering ferroptosis [17, 4, 18]. We therefore investigated the effect of mTOR inhibition on these features after 48 h of treatment. Firstly, Sal treatment markedly increased ROS levels, including both global ROS and hydroxyl radicals, as well as iron levels, as expected; while co-treatment with Torin significantly reduced ROS and iron levels (Fig. 2B–D). The level of lipid peroxidation was not significantly impacted by any of the treatments (Fig. S2A). Surprisingly, the siRNA treatments had no effect on ROS and iron levels induced by Sal (Fig. S2B and S2C). Meanwhile, as expected, iron chelators (including iron chelator IV and deferoxamine (DFO)) affected Sal–induced ferroptosis. More importantly, iron supplementation with FeCl_3_ is sufficient to affect the mTOR inhibition-mediated resistance to the Sal–induced ferroptosis (Figs. S2D and E).

To determine the mechanisms by which Torin protects against Sal-induced iron overload, iron entry was first considered in cells treated during 48 h. Confirming previous observations [4], Sal elicited iron uptake via increased transferrin (Tf)-Uptake and CD71 expression at the cell surface (Fig. 2E, F) as well as at the total protein (Fig. 2G quantifications in S2F) and mRNA (Fig. S2G) levels. In contrast, Torin alone, or in combination with Sal, prevented iron uptake (Fig. 2E–G). Cellular iron content also results from the degradation of ferritin, an iron storage molecule, which is recycled/degraded in lysosome under low iron levels, namely ferritinophagy [19] (Fig. 2A). Consistent with our previous report [4], Sal triggered ferritinophagy as shown by decreased FTH protein level (Fig. 2G – quantification in S2H) and increased FTH mRNA level (Fig. 2H). Remarkably, although Torin is a strong inducer of autophagy and inhibitor of protein synthesis, co-treatment with Torin upregulated the level of FTH protein (but not its mRNA) compared to Sal alone (Fig. 2G, H – quantification in S2H). To further investigate ferritin degradation, cells were additionally treated with a specific autophagy inhibitor (Hydroxychloroquine, HCQ) [20], as confirmed by the accumulation of p62 and LC3 proteins (Fig. S2I). HCQ and Sal co-treatment induced an increase in FTH protein level compared to Sal alone. However, it did not affect Sal-induced cell death (Figs. S2I and S2J), suggesting that blocking FTH degradation is not sufficient to prevent cell death. Co-treatment of HCQ with Torin (with or without Sal) did not increase the FTH protein level compared to Torin (with or without Sal), indicating that mTOR inhibition per se prevents the proteostasis of FTH (Fig. S2I). Overall, these data indicate that mTOR inhibition prevents ROS production and iron homeostasis dysregulation induced by Salinomycin.

### Salinomycin drastically disrupts the expression of mitochondrial proteins which is protected by mTOR inhibition

**Fig. 3:**
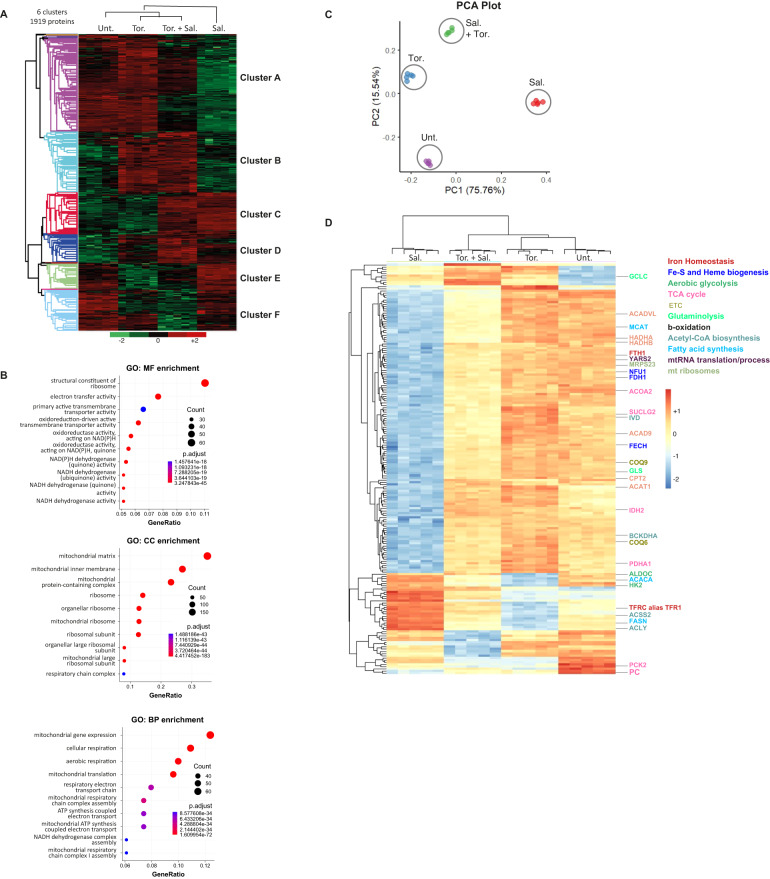
Salinomycin drastically impairs the expression of mitochondrial proteins which is protected by mTOR inhibition. HMLER CD24L cells were treated or not with Sal (500 nM) or/and Tor. (250 nM) for 48 h. **A** Unsupervised clustering performed with the 1919 proteins differently modulated between the different conditions of treatment from proteomic data (ANOVA test, FDR < 0.01, S0 = 1). 6 main distinct clusters have been identified. Each column is a sample; each row a protein. The color scale indicates the protein expression value (green: lowest; red: highest). The intensity of each protein corresponds to the relative abundance of individual proteins by liquid chromatography-mass spectrometry (nanoLC-MS/MS). Proteins were clustered using Perseus software with Euclidean distances. (*n* = 5 replicates). **B** Dot plot of the best Gene Ontology (top 10 terms regarding best adjusted p-value) for Molecular Function (MF), for Cellular Component (CC), and for Biological Process (BP) obtained from the differentially expressed proteins of cluster A. The size of the circles represents the number of proteins (Count) found enriched for each corresponding term. **C**, **D** Identification of a 187-protein-based discriminant signature of the four experimental conditions investigated by proteomic analysis. **C** Principal component analysis performed with the 187-protein based signature supervised by machine learning. **D** Unsupervised clustering performed with the 187-protein based signature.

To further investigate the key actors involved in Sal action and also in ferroptosis suppression by mTOR inhibition, we carried out a proteomic analysis by using mass spectrometry on cells treated for 48 h. In total, we identified and quantified 6396 proteins. Among them, the levels of 1919 proteins were found to be significantly different after 1-way ANOVA analysis (FDR 1% and s0 = 1) between the four conditions (Table S1), and an unsupervised hierarchical clustering analysis identified 6 main clusters. Cluster A showed proteins downregulated by Sal treatment compared to control, and upregulated by co-treatment with Torin compared to Sal alone (Fig. 3A). Functional annotation of this cluster using molecular function (MF), cellular component (CC), and biological process (BP) terms pointed to mitochondrial function and ribosomal activity (Figs. 3B and S3).

To determine the key pathways involved in ferroptosis protection through mTOR inhibition, a minimal proteomic signature of 187 proteins was identified by processing a supervised machine learning on the 1,919 proteins differentially expressed (Table S2). All the steps of the analysis are detailed in Materials and Methods and illustrated in Figure S4A–C. By unsupervised principal component analysis, the 187-proteins signature successfully discriminated the samples on the first two principal components accounting for more than 91% of the signature variance (Fig. 3C). Unsupervised hierarchical clustering analysis of proteomic data allowed to discriminate sample groups according to the expression of these 187 proteins (Fig. 3D). Most of the proteins contained in this signature are downregulated by Sal treatment (150/187) as compared to control, of note, their levels are upregulated upon co-treatment with Torin (Fig. 3D). Among the signature, we found proteins involved in iron homeostasis, including FTH and TfR, which are oppositely regulated by Sal alone versus co-treatment with Torin, (Fig. 3D – proteins written in dark red) in agreement with our previous results. Furthermore, proteins involved in mitochondrial iron metabolism and essential for mitochondrial functions, including Fe-S or heme biogenesis, were found to be downregulated by Sal, and in comparison, up-regulated by co-treatment (Fig. 3D – proteins written in blue). Once again, this data highlights that the iron dysregulation induced by Sal is attenuated by co-treatment with Torin. Besides, functional enrichment analysis performed with KEGG database on the network reported a strong enrichment in metabolic pathways associated with mitochondrial pathways (Fig. S4D, E). In line with this, we also found in this signature many proteins involved in mitochondrial -pathways or -related pathways (e.g., TCA cycle, electron transport chain, glycolysis and glutaminolysis, β-oxidation) and in mitochondrial biogenesis (e.g., mtRNA translation/process and in mt ribosomes) as highlighted in Fig. S5. Considering the importance of iron metabolism in mitochondrial function, this suggests that in our model iron dysregulation drives mitochondrial alterations and triggers ferroptosis.

### Integration of -omics data highlighted a metabolic shift under Sal treatment, prevented by mTOR inhibition

**Fig. 4:**
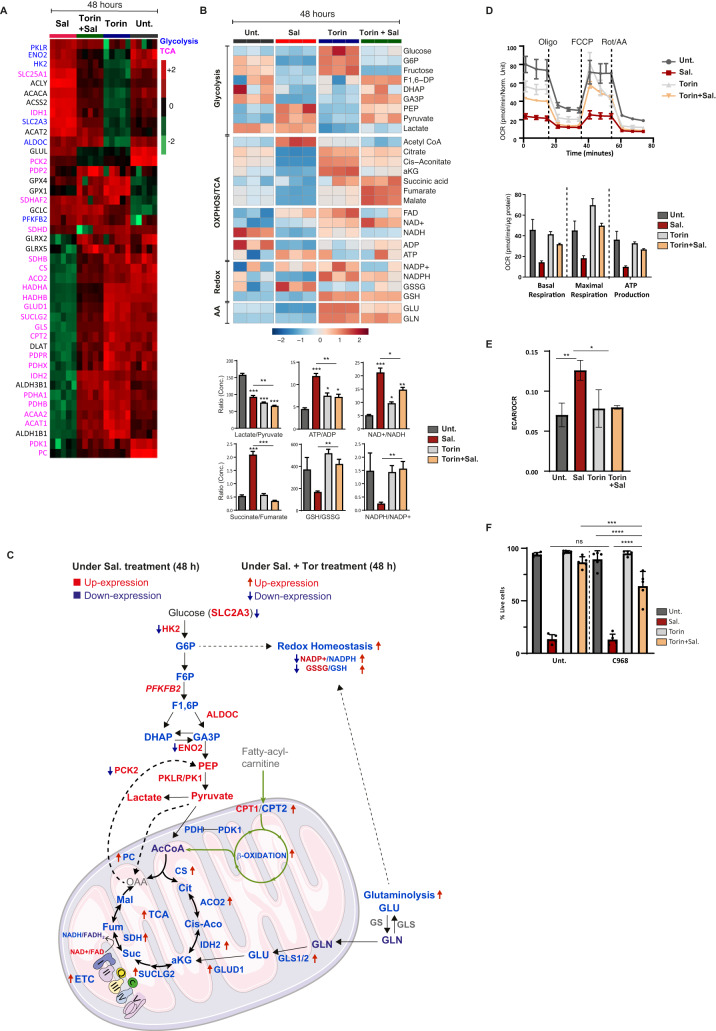
mTOR inhibition interferes with Sal-mediated enhanced aerobic glycolysis and restores the mitochondrial-associated metabolic pathways. **A** Heatmap comparing relative levels of proteins related to TCA cycle (written in pink) and glycolysis (written in blue) in HMLER CD24L cells in response to Sal, or Tor. alone, and combination treatments for 48 h. Color key indicates protein expression value (green: lowest; red: highest). Proteins were clustered using Perseus software. **B** Upper: Heatmap comparing relative levels of metabolites related to OXPHOS or TCA cycle and to glycolysis and also to glutaminolysis in HMLER cells in response to Sal, or Tor. alone, and combination treatments for 48 h. Color key indicates metabolite expression value (blue: lowest; red: highest). Metabolites were clustered using Metaboanalyst software. *Lower:* Graphs represent the mean of lactate/pyruvate, ATP/ADP, NAD + /NADH, Succinate/Fumarate ratios indicating the use of glycolysis or OXPHOS. GSH/GSSG and NADPH/NADP+ ratios are indicators of oxidative stress. Two tailed and unpaired student’s *t* test. *p < 0.05; **p < 0.01; ***p < 0.001. **C** Summary of the differential level of metabolites and proteins associated with the OXPHOS/TCA cycle, with glycolysis, with b-oxidation, with glutaminolysis, and redox homeostasis under Sal treatment (terms) and under combination treatment (arrows). Blue and red colors indicate the downregulated and upregulated levels, respectively. **D** Seahorse-based measurements of OCR (*upper panel*) in HMLER CD24L cells incubated under treatments as indicated for 48 h, normalized to total protein levels. Upper panel: Oligomycin, carbonyl-cyanide-4-(trifluoromethoxy)phenylhydrazone (FCCP), and antimycin A were serially injected to measure ATP production, maximal respiration, and basal respiration, respectively, as indicated in the graph. **E** Basal ECAR/OCR ratio in HMLER CD24L cells treated as indicated for 48 h. **F** HMLER CD24L was treated during 96 h with either Sal (500 nM), Torin (250 nM), or a combination of both in the presence or in the absence of glutaminolysis inhibitor (C968 (20 μM)). Cell death was determined by dapi staining and flow cytometry. *n* = 3 independent experiments (with at least duplicate). Data are presented as: mean ± SD, ANOVA test: *p < 0.05; **p < 0.01; ***p < 0.001; ****p < 0.0001.

Ferroptosis has been described as a metabolism-associated cell death [21], and given the overall impact observed on mitochondrial function, we postulated that the protective effect of mTOR inhibition could be mediated by changes in cellular metabolism. To this end, a targeted analysis of individual metabolites using liquid chromatography–mass spectrometry (LC-MS) was performed on cells treated for 48 h. In order to identify the key pathways involved not only in the execution of ferroptosis by Sal but also those leading to its blockage by Torin, we decided to integrate metabolomics and proteomics data (Fig. 4). Figure 4A, B show the heatmap of proteins and metabolites levels involved in mitochondrial pathways (OXPHOS/TCA) and mitochondrial-related pathways (glycolysis and glutaminolysis) in cells treated for 48 h. Consistent with the reduced levels of TCA- and ETC- related enzymes, Sal treatment dramatically reduced the levels of the majority of TCA cycle intermediates, with an increase in acetyl-Coenzyme A (acetyl-CoA) indicating impaired TCA activity (Fig. 4A proteins in pink and Fig. 4B). In addition, the accumulation of succinate shown by the increased succinate/fumarate ratio and the impaired level of Succinate Dehydrogenase SDH (or complex II), supports this Sal-mediated TCA- and ETC- alteration (Fig. 4B, lower panel). Therefore, as summarized in Fig. 4C, Sal treatment inhibits both TCA and ETC pathways, yet the addition of Torin seems to restore both pathways.

Next, regarding glycolysis, we found that Sal treatment (compared to untreated) decreased the lactate-to-pyruvate ratio (anaerobic glycolysis marker) and increased the ATP/ADP ratio (Fig. 4B, lower panel), as well as upregulated cytoplasmic glycolytic enzymes (Fig. 4A proteins in blue). In contrast, the pyruvate dehydrogenase complex (PDH) that converts pyruvate into Acetyl-CoA for TCA cycle is downregulated (Fig. 4B, lower panel). To go a step further, we assessed mitochondrial and glycolytic activity by measuring the oxygen consumption rate (OCR) and the extracellular acidification rate (ECAR) respectively using Seahorse-based assays. (Fig. 4D). We confirmed Sal-induced dysregulation of ETC with significant alteration of both basal and maximal respiration and ATP production, all prevented by co-treatment with Torin (Fig. 4D). Besides, the basal ECAR-to-OCR ratio increased under Sal treatment, demonstrating higher glycolytic compared to mitochondrial activity, which was reversed under Torin treatment (Fig. 4E). In summary, Sal-treated cells shift to higher aerobic glycolysis to produce ATP, while co-treated cells restore the expression of TCA-associated enzymes, ETC-related proteins and OXPHOS-mediated ATP production (Fig. 4C).

### Activation of glutaminolysis seems to drive ferroptosis suppression by mTOR inhibition

Apart from glycolysis, TCA can also be sustained by glutamine anaplerosis (Fig. 4C). Along with the decrease in TCA activity, Sal treatment also reduced the levels of glutaminolysis-related metabolites and enzymes (GLS, GDH, and GLUD1) (Fig. 4A, B). In addition, glutamine is also involved in redox-homeostasis through the production of GSH and NADPH, two essential substrates for the antioxidant defenses. Sal treatment resulted in a decrease in the NADPH-to-NADP+ ratio and a decrease in reduced-to-oxidized glutathione ratio (GSH/GSSG) (Fig. 4B, lower panel). Importantly, all these modulations were restored upon co-treatment with Torin, suggesting increased glutaminolysis and improved oxidative stress management, consistent with our previous results. To determine whether activation of glutaminolysis contributes to Torin protection against ferroptosis, cells were additionally treated with the glutaminase (GLS) inhibitor: Compound C968 (C968). Co-treatment with C968 did not affect Sal-induced cell death (Fig. 4F). However, it did sensitize cells to Sal-induced cell death upon co-treatment with Torin. Therefore, inhibition of glutamine anaplerosis prevents Torin treatment from suppressing Sal-induced cell death.

As summarized in Fig. 4C, Sal-treated cells undergo a profound metabolic reprogramming which could result in a decrease in redox homeostasis. On the contrary, upon mTOR inhibition, cells showed greater oxidative metabolism, with increased TCA- and ETC- activity, as well as overactivation of glutaminolysis that contributes to Torin-induced defense against ferroptosis.

### mTOR inhibition overcomes salinomycin-induced mitochondrial dysfunction

**Fig. 5:**
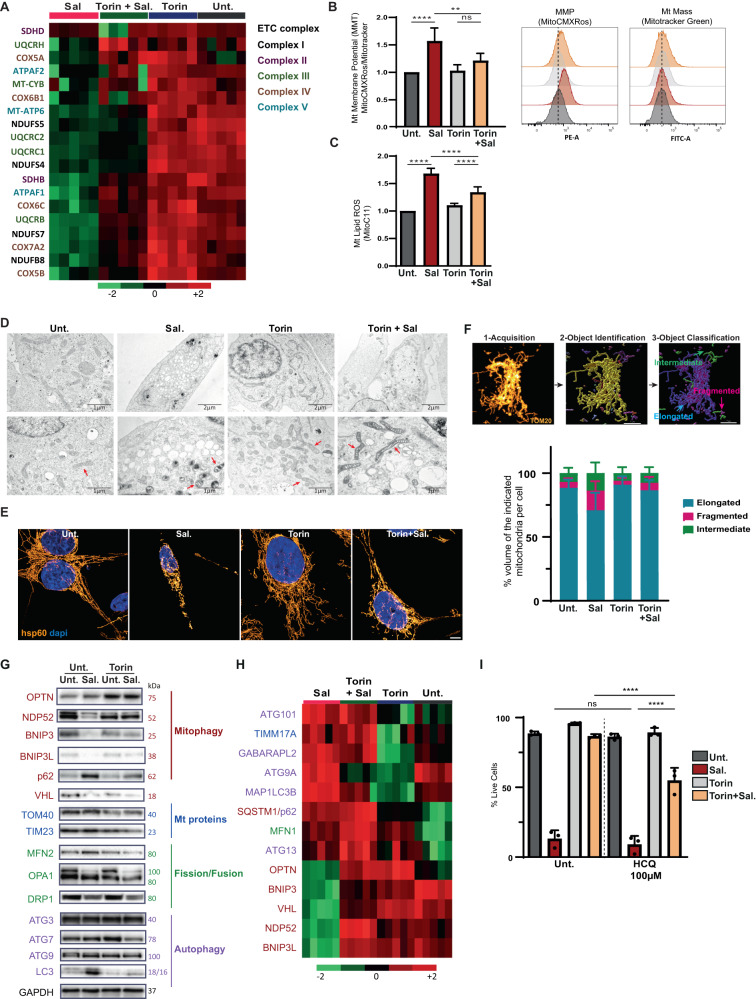
mTOR inhibition prevents mitochondrial damages induced by Sal. HMLER CD24L were treated with either Sal, Torin, or a combination of both for 48 h. **A** Heatmap of ETC proteins from proteomics data. Color key indicates protein expression value (green: lowest; red: highest). **B** Mitochondrial membrane potential was measured with MitoCMXRos and Mitotracker Green staining. Data are shown as a ratio of MitoCMXRos over Mitotracker Green (*n* = 5). **C** Mitochondrial lipid peroxidation was measured by Mito-C11 probe staining coupled with FC. Representative histogram of oxidized-Mito-C11 (FITC channel) intensity level in cells (*n* = 3). **D** TEM images, red arrows show mitochondria. **E** Immuno-staining of mitochondria with hsp60 and images acquired using Confocal Leica TCS SP5. Objective: 63×. Scale bar: 5 μm. **F** Upper panel, steps of the workflow developed to analysis mitochondrial network: first, a machine learning (ML) identified mitochondria as objects, then a second ML classified mitochondria into three classes depending on their shapes: fragmented, intermediary, and hyperfused. Scale bar: 7 μm. *Lower panel*, Quantification of mitochondrial network analysis, data shown as % area of each class for each cell. Analysis of>25 cells from 2 independent experiments. **G** Immunoblotting for the indicated autophagic/mitophagic proteins and Mt/dynamic proteins. GAPDH was used as the loading control. **H** Heatmap of autophagic/mitophagic proteins and Mt/dynamic proteins from proteomics data. Color key indicates metabolite expression value (green: lowest; red: highest). Protein terms and functions indicated with the same color are corresponding for (**G**) and (**H**). **I** HMLER CD24L cells were treated with Sal or/and Tor. in the presence or in the absence of HCQ (100 μM) for 144 h. Cell death was determined by dapi staining coupled with flow cytometry (FC). The graph represents the mean ( ± SEM) of three independent experiments. One-way ANOVA test. *p < 0.05; **p < 0.01; ***p < 0.001; ****p < 0.0001. ns, not significant.

Given that Sal-treatment massively affects mitochondria-related processes, we sought to better understand how the different treatments impinge on mitochondria (Fig. 4C). Similarly, with respect to mitochondrial respiration, the treatment decreased the protein levels of the complexes I, II, III, and IV of the ETC, whereas co-treatment with Torin prevented this reduction (Fig. 5A, S6A - quantification in S6B). For further investigation, mitochondrial membrane potential (ΔΨ) (generated by complexes I, III, and IV) was measured using mitochondrial probes: MitoCMXRos accumulates in negatively charged mitochondria and MitroTracker accumulates in the matrix. By analyzing the ratio of MitoCMXRos-to-MitoTracker, we found that Sal treatment increased ΔΨ, while co-treatment with Torin reduced it (Fig. 5B). Interestingly, mitochondrial mass was unaffected by the treatments (Fig. 5B, right panel). Defective mitochondria are more likely to produce ROS that could promote lipid peroxidation. Therefore, lipid-ROS levels were measured using a mitochondria-targeted fluorescence lipid peroxidation probe (MitoPerOx) [22]. Sal treatment alone increased Mt lipid peroxidation, while co-treatment with Torin reduced it (Fig. 5C). Taken together, these data indicate that mTOR inhibition prevents Sal-mediated mitochondrial dysfunction and oxidative stress.

### Salinomycin profoundly alters the mitochondrial network while mTOR inhibition restores a reticular network

Since mitochondrial activity is altered by Sal treatment, we qualitatively evaluated the mitochondrial network during treatment. Firstly, an analysis of mitochondria ultrastructure by transmission electron microscopy (TEM) revealed that Sal- treated cells had smaller mitochondria with profound alteration in their structural integrity such as darker matrix and irregular cristae compared to untreated cells (Fig. 5D - red arrows show mitochondria). On the contrary, cells treated with Torin showed much less affected mitochondria, with enlargement of mitochondrial cristae. Analysis of the mitochondrial network by immunostaining with a mitochondrial matrix protein (hsp60, Heatshock protein 60) confirmed that Sal treatment significantly affected the mitochondrial network with fragmented mitochondria, while a phenotype close to untreated cells was observed in co-treated cells (Fig. 5E). To take this step further, a workflow for the analysis of 3-dimensional mitochondrial network was set up as described precisely in the Materials and Methods section. Figure 5F*upper panel,* briefly details the workflow steps. This analysis highlighted the increased proportion of fragmented mitochondria in Sal-treated cells compared with untreated, Torin- or co-treated cells (Fig. 5F, lower panel and S6C). Overall, these data indicate that mTOR inhibition prevents the profound alteration in the mitochondrial morphology induced by Salinomycin.

### mTOR inhibition prevents mitochondrial dynamic alteration induced by salinomycin

The maintenance of the mitochondrial network is a highly dynamic process regulated by fusion and fission events, as well as the elimination of damaged mitochondria, a process named mitophagy [23]. Since Torin is known to activate mitophagy [24], and given the impact of treatment on the mitochondrial network, we decided to study key actors involved in mitochondrial dynamics. Proteomic data as well as western blot analysis showed downregulation of several mitophagy proteins (including BNIP3/3 L, OPTN, NDP52) in Sal-treated cells, whereas Torin treatment restored it (Fig. 5G, H). Besides, as described in our previous work [25], although ATG initiator proteins were upregulated, an accumulation of LC3-II and p62 was observed following Sal treatment indicating an inhibition of autophagic flux that is restored upon co-treatment. Then, mitochondrial DNA (mtDNA), cleared during mitophagy to avoid accumulation, was measured by qPCR [26]. Co-treatment decreased the mitochondrial mtDNA mass as compared to Sal alone, (Figure S6D) supporting the activation of mitophagy in co-treated cells.

In line with these results, TEM image acquisition revealed that cells co-treated with Torin exhibited advanced autophagic degradative vacuoles/autolysosomes containing damaged organelles, including a structure with the same dark density as the damaged mitochondria (Fig. S6E). We postulated that, under Salinomycin treatment, early stages of mitophagy occur, but the late stages could be blocked, leading to an accumulation of damaged mitochondria. Besides, mitochondrial biogenesis is inhibited during Sal treatment (in view of the decrease in the protein levels of TFAM and others including POLRMT, TFB2M, mTERF). To go further, we explored the interaction between mitochondria and lysosomes by immunofluorescence microscopy (Fig. S6f). Interestingly, although we observed that mitochondrial mass measured by Mitotracker (Fig. 5B) was not significantly affected in Sal-treated conditions, we revealed here that while the lysosomal area was not affected, Salinomycin alone or in combination with Torin significantly increased the mitochondrial area. More importantly, the results showed that interactions between mitochondria and lysosomes (measured as the percent of colocalization) were significantly reduced under Salinomycin treatment compared with control. In contrast, Torin treatment in combination with salinomycin restored mitochondria-lysosome interaction levels to control. These data therefore support that the interactions between lysosomes and mitochondria are impaired by Sal-treatment and restored when combined with Torin. To go a step further, the importance of Torin-induced clearance of damaged mitochondria in the protection against ferroptosis was examined by treating cells additionally with HCQ (100 μM) to block mitophagy [27, 28]. HCQ efficiently inhibits the late stage, i.e. the fusion of mitophagosomes with lysosomes, and blocks the degradation of mitochondria. We addressed by western blot analysis whether treatment with HCQ restored the levels of NDP52, BNIP3, and OPTN levels under Salinomycin treatment. The results showed that HCQ was sufficient to restore the expression of these proteins under salinomycin and also to increase their expression under other conditions (Fig. S6G). These data indicate that Salinomycin effectively targets their lysosomal degradation. However, the expression of TIM23, used as an indicator of the mitochondrial mass, did not increase under Sal in combination with HCQ compared with Sal alone. In contrast, TIM23 expression decreased under Torin treatment, in combination or not with Sal, compared with untreated or Sal-treated cells. Under these Torin treated conditions, HCQ increases TIM23 expression. These data suggest that although Salinomycin targets the degradation of well-known mitophagic receptors (including NDP52, BNIP3, and OPTN), the mitochondrial mass is not decreased, indicating, as described above, that the early stages of mitophagy occurred, but that the later steps could be blocked, leading to an accumulation of damaged mitochondria. Thus, Torin could be able to (re-)activate (the late stages of) mitophagy to overcome the accumulation of damaged mitochondria. HCQ treatment after 144 h significantly decreased the viability of co-treated cells, restoring their sensitivity to Sal-induced cell death (Fig. 5I). Of note, HCQ alone or with Torin did not affect the Sal-induced ferroptosis. Taken together, these results suggest that autophagic removal of Sal-induced damaged mitochondria may contribute to the restoration of mitochondrial function and protection from ferroptosis induced by mTOR inhibition.

## Discussion

**Fig. 6:**
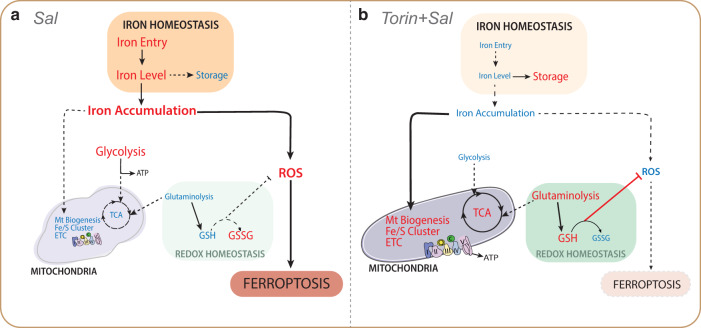
Schematic proposal model of the suppression of ferroptosis by mTOR inhibition. **A** Pathways regulated upon Sal treatment compared to control. Mechanistically, Sal induces a burst of iron and ROS, and triggers a metabolic shift by decreasing the level of mitochondrial proteins and the activity of mitochondrial metabolic pathways. Besides, Sal inhibits redox homeostasis, which leads to even more ROS production, and subsequently triggers ferroptosis. **B** Pathways regulated upon co-treatment with Torin and Sal compared to Sal alone. In contrast, inhibition of mTOR prevents the Sal-induced accumulation of iron and ROS, as well as the functional and structural alteration of mitochondria. Besides it activates glutaminolysis to improve redox defenses which leads ultimately to the inhibition of ferroptosis. Blue and red colors indicate the downregulated and upregulated level, respectively.

In the present study, we examined the key role of the mTOR pathway in ferroptosis induced by Salinomycin. In particular, mTOR inhibition interferes with Sal-induced iron dysregulation and metabolic rewiring, thereby decreasing the sensitivity of CSCs to ferroptosis, and notably preserving the integrity of the mitochondrial network and promoting the clearance of damaged mitochondria (Fig. 6).

The accumulation of iron within cells through Sal-induced increased iron entry is impaired by mTOR inhibition, consistent with the work of Bayeva et al. [29] showing that mTOR inhibition decreases the stability of TfR1 mRNA and alters cellular iron flux. This suggests that fewer Fe^2+^ ions are mobilized for Fenton chemistry, consistent with the reduced ROS level. Altogether, these data indicate that mTOR inhibition affects various molecular regulators related to iron homeostasis. Metabolomic and proteomic analyses highlighted mitochondria as a key regulator of Sal-induced ferroptosis, supporting the numerous studies showing mitochondria to be a major hub of ferroptosis [30–32]. Interestingly, Smethurst et al., recently identified that iron promotes ribosome RNA degradation under oxidative stress in yeast [33]. Besides, ROS accumulation impairs the mitochondria integrity including mtDNA maintenance and mtRNA processing [34], and inhibition of Sal-induced accumulation of mtROS under mTOR inhibition, may limit the severe mitochondrial damages, notably by increasing de novo GSH synthesis [13].

Furthermore, some recent data have shown that treatment with a Sal-derivative termed AM5, leads to a decrease in mitochondrial iron level in acute myeloid leukemia [35]. Interestingly, the expression of Frataxin (FXN), a protein involved in Fe/S cluster biogenesis, has been recently identified as a negative regulator of ferroptosis, and is upregulated upon mTOR inhibition [36]. The effect of Sal on mitochondrial dysfunction is likely mediated by certain downregulated Fe/S proteins, which are upregulated with co-treatment. Particularly, NEET proteins including CISD1 (also termed mitoNEET) and CISD3 (also known as Mine2/MiNT) (which regulate iron and ROS homeostasis) have been demonstrated to protect cells from mitochondrial damage in ferroptosis [37, 38]. Among Fe/S-containing enzymes, loss of Succinate Dehydrogenase (SHDB/C) (protein related to TCA/ETC), has been shown to affect iron homeostasis and promote ferroptosis [39]. Thus, Salinomycin drastically induces a profound metabolic reprogramming toward aerobic glycolysis, which is characterized by the downregulation of mitochondrial metabolic pathways related to TCA cycle/ETC activity. In agreement with a previous report [40], inhibition of mTOR could also restore TCA cycle activity by anaplerosis, notably via glutaminolysis, which could also be involved in the maintenance of redox homeostasis through de novo GSH synthesis, as described above.

We revealed by machine learning analysis of the mitochondrial network that Sal increases intermediate and fragmented mitochondria, while cells treated with the mTOR inhibitor display a tubular mitochondrial network. However, the dysregulation of mitochondrial dynamics proteins induced by Sal treatment does not *fit* with the fragmented networks observed, raising the question: by which mechanisms? One possible explanation is the impact of redox-sensitive post-translational modifications on their activity. For example, it has been shown that only the mature L-isoform (L ~ 100 kDa vs S ~ 80 kDa) of the dynamin-like GTPase OPA1 has a mitochondrial fusion stimulating activity [41]. Or again, that under oxidative stress S-nytrosylation increases the GTPase activity of DRP1 [42]. Besides, mitochondrial membrane hyperpolarization upon Sal treatment may contribute to enhanced mitochondrial fission. In line with this, we found that inhibition of mitophagy contributes to restoring cell sensitivity to Sal-cell death upon mTOR inhibition. Interestingly, NDP52 has been recently identified as a redox sensor in autophagic clearance of damaged mitochondria [43]. Our results show that Sal treatment dramatically affects mitochondrial dynamic and function, and may overcome the cell’s capacity to clear damaged mitochondria even under massive oxidative stress, mechanisms which are, in several ways, prevented by mTOR inhibition. These data are in agreement with other studies showing that mTOR inhibition protects against mitochondrial diseases through mitophagy activation [44–46].

As mTOR inhibitors in monotherapy have been shown to be effective in several types of cancer, numerous clinical trials have explored the potential of mTOR inhibitors in combination with other molecularly-targeted or chemotherapeutic agents to reverse drug resistance [47–50]. Besides, while mTOR inhibitors have been reported to suppress CSCs [51], other studies have demonstrated their ability to induce the expansion of drug-resistant CSCs, notably in breast and colorectal cancer [52, 53]. It appears that the effects of mTOR inhibitors on CSCs may be context or cell type dependent. Interestingly, induction of ferroptosis is now a novel approach to overcome drug or immunotherapy resistance [54], as shown in preclinical studies. Therefore, our work highlights that the metabolic status of the cell driven by the mTOR pathway can modulate the sensitivity of breast CSCs to Sal-induced ferroptosis. Finally, it provides proof-of-concept that careful evaluation of such combination therapy (here co-targeting mTOR inhibition and ferroptosis) is required to develop effective treatments.

## Methods

### Cell line and culture

The human mammary epithelial cell line infected with a retrovirus carrying hTERT, SV40, and the oncogenic allele HrasV12, referred to as HMLER CD24L cells, is a subclone known to be rich in the ‘stemness’ phenotype [7]. HMLER CD24L cells were a generous gift from A. Puisieux (INSERM 1052, Lyon, France). HMLER CD24L cells were cultured in DMEM/F12 + GlutaMAX (Gibco, 31331) supplemented with 10% Fetal Bovine Serum (FBS, Eurobio, CVFSVF00-01), 10 μg/mL Insulin (Sigma, I9278) 0.5 μg/mL hydrocortisone, 10 ng/mL human EGF (Peprotech, AF-100-15), and 0.5 μg/mL puromycin (Invivogen).

### Drugs treatments

Unless specified otherwise, cells were seeded at a density of 40,000 cells/well in the medium in 12-well plates (or at a density of 1 × 10^4^ cells/well in 6-well plate for western blot and RT-qPCR). At 70–80% confluence (2–3 days after) cells were treated with the indicated treatments. Drugs are listed in Supplementary Table S3.

### Cell death (Flow Cytometry)

Cells were treated as indicated during the desired time, then detached with trypsin, collected, and rinsed with PBS. Subsequently, DAPI (1:2000 in PBS) was added for dead cell quantification using a flow cytometer (Fortessa, BD Biosciences). A minimum of 50,000 cells was analyzed per condition. Necrotic/late or early apoptotic events were evaluated by annexin V labeling using the annexin V-FITC / Propidium Iodide (PI) assay kit (556570, BD Pharmingen^TM^) according to the standard protocol.

### Cell death (IncuCyte)

Cells were seeded in 24-well plates at a density of 20,000 cells by well and treated with the indicated treatments. Simultaneously, NucLight Rapid Red probe (4717, Essen BioScience) and Cytotox Green probe (4633, Essen BioScience) were added. Images (20X) of the same fields were taken every 2 h for 96 h by a real-time IncuCyteS3 Live-Cell analysis system (Essen Bioscience, Ann Arbor, Michigan, USA). Fluorescence intensities were analyzed using the IncuCyte software, and results were displayed normalized to the initial time point (time *t* = 0).

### Measurement of Fluorescent probes staining

Cells were treated as indicated during the desired time, then detached with trypsin, collected and rinsed with PBS. Subsequently, probes diluted in HBSS were added (with the indicated concentration) and incubated during 30 mn (except if specified) at 37 °C + 5% CO_2_. Excess probed were removed by washing the cells with PBS, and DAPI (1:2000) was added to stain dead cells. Fluorescence signals were measured using a flow cytometer (Fortessa, BD Biosciences). A minimum of 50,000 cells were analyzed for each condition. Data were processed using BD FACSDiva software and analysed using FlowJO software on median fluorescence level gated on live cells. The probes (with their concentration and their incubation time if different from 30 mn) are listed in Supplementary Table S3.

### Proteomics

#### Sample preparation for proteomic analysis

S-TrapTM micro spin column (Protifi, Huntington, USA) digestion was performed on 20 µg of cell lysates according to manufacturer’s instructions and optimized as described in Ceccacci et al., 2022 [55]. After elution, peptides were vacuum-dried and resuspended in 50 µl of 2% ACN, 0.1% FA, and quantified by Nanodrop [55].

#### nanoLC-MS/MS protein identification and quantification

400 ng of each sample was injected on a nanoElute (Bruker Daltonics, Germany) HPLC (high-performance liquid chromatography) system coupled to a timsTOF Pro (Bruker Daltonics, Germany) mass spectrometer. HPLC separation (Solvent A: 0.1% formic acid in water; Solvent B: 0.1% formic acid in acetonitrile) was carried out at 250 nL/min using a packed emitter column (C18, 25 cm × 75 μm 1.6 μm) (Ion Optics, Australia) using gradient elution (2 to 13% solvent B during 41 min; 13 to 20% during 23 min; 20% to 30% during 5 min; 30% to 85% for 5 min and finally 85% for 5 min to wash the column). Mass-spectrometric data was acquired using the parallel accumulation serial fragmentation (PASEF) acquisition method. The measurements were carried out over the m/z range from 100 to 1700 Th. The range of ion mobilities values from 0.75 to 1.25 V s/cm^2^(1/k0). The total cycle time was set to 1.17 s and the number of PASEF MS/MS scans was set to 10.

#### MS data processing and bioinformatics analysis

The obtained data were analyzed using MaxQuant version 2.0.1.0 and searched with Andromeda search engine against the UniProtKB/Swiss-Prot *Homo sapiens* database (release 02-2021, 20408 entries). To search parent mass and fragment ions, we set a mass deviation of 3 ppm and 20 ppm respectively. The minimum peptide length was set to 7 amino acids and strict specificity for trypsin cleavage was required, allowing up to two missed cleavage sites. Carbamidomethylation (Cys) was set as a fixed modification, whereas oxidation (Met) and N-term acetylation were set as variable modifications. The false discovery rates (FDRs) at the protein and peptide levels were set to 1%. Scores were calculated in MaxQuant as described previously [56]. The reverse and common contaminants hits were removed from MaxQuant output. Proteins were quantified according to the MaxQuant label-free algorithm using LFQ intensities; protein quantification was obtained using at least 1 peptide per protein. Match between runs was allowed.

Statistical and bioinformatic analysis, including heatmaps, profile plots, and clustering, were performed with Perseus software (version 1.6.15.0) freely available at [57]. Intensities were log2 transformed for statistical analysis. For statistical comparison, we set 4 groups, each containing 5 biological replicates. We then filtered the data to keep only proteins with at least 5 valid values in at least one group. Next, the data were imputed to fill missing data points by creating a Gaussian distribution of random numbers with a standard deviation of 33% relative to the standard deviation of the measured values and 1.8 standard deviation downshift of the mean to simulate the distribution of low signal values. We performed an ANOVA test, FDR < 0.01, S0 = 1. Hierarchical clustering of proteins that survived the test was performed in Perseus on logarithmised LFQ intensities after Z-score normalization of the data, using Euclidean distances.

### Targeted liquid chromatography mass spectrometry analysis of metabolites

For targeted metabolomics analysis, 2 × 10^5^ WT and KD.7 cells were treated or untreated with 100 ng/mL OSM for 24, 48, and 72 h. Each sample was washed three times with cold DPBS, frozen in liquid nitrogen, and stored at −80 °C. Metabolites were extracted with an adequate volume (calculated from cell count 2 × 10^6^ cells/mL) of an aqueous solution of methanol and acetonitrile (20:50:30). Samples were vortexed for 5 min at 4 °C and then centrifuged at 16,000 g for 15 min at 4 °C. The supernatants were collected and stored at −80 °C until analysis. LC/MS analyses were conducted on a Q Exactive Plus Orbitrap mass spectrometer equipped with an Ion Max source and a HESI II probe and coupled to a Dionex UltiMate 3000 UPLC system (Thermo Fischer Scientific). External mass calibration was performed as recommended by the manufacturer. A 5 µL aliquot of each sample was injected onto a ZIC-pHILIC column (150 mm × 2.1 mm i.d. 5 μm, Millipore) fitted with a guard column (20 mm × 2.1 mm i.d. 5 μm, Millipore) for the liquid chromatography separation. The chromatographic gradient was run at a flow rate of 0.200 μl/min with Buffer A (20 mM ammonium carbonate and 0.1% ammonium hydroxide, pH 9.2) and Buffer B (acetonitrile) as follows: at 0–20 min, a linear gradient from 80% to 20% B was administered; at 20–20.5 min, a linear gradient from 20% to 80% B was administered; at 20.5–28 min, 80% B was maintained. The mass spectrometer was operated in full-scan, polarity-switching mode, with the spray voltage set to 2.5 kV and the heated capillary held at 320 °C. The sheath gas flow was set to 20 units, the auxiliary gas flow was set to 5 units, and the sweep gas flow was set to 0 units. The metabolites were detected across a mass range of 75–1000 m/z at a resolution of 35,000 (at 200 m/z), with the AGC target at 10^6^ and the maximum injection time at 250 ms. Lock masses were used to ensure a mass accuracy below 5 ppm. Data were acquired with ThermoXcalibur software. The peak areas of the metabolites were determined using Thermo Trace Finder software and identified by the exact mass of each singly charged ion and by the known retention time on the HPLC column. Statistical and pathway analyses were performed using Metaboanalyst 5.0 software.

### Measurement of OCR and ECAR

OCR and ECAR were analyzed using the Seahorse XFe96 bioenergetic analyzer in accordance with the manufacturer’s instructions (Agilent Technologies). Briefly, HMLER CD24^low^ were seeded at a density of 10^4^ cells per well in a specialized 96-well Seahorse XF96 V3 PS microplate (101085-004, Agilent Technologies). 48 h after, cells were incubated with 500 nM Salinomyin and/or 250 nM of Torin. Cells were incubated for 1 h in unbuffered XF assay media (Agilent Technologies) supplemented sequentially with either 2 mM glutamine, 25 mM glucose (G8769, Sigma Aldrich), and 1 mM sodium pyruvate for OCR analysis. For OCR/ECAR measurements, the XF Cell Mito Stress Test (103015-100, Agilent Technologies) was used. Compounds were injected during the assay at the following final concentrations: oligomycin (75351, Sigma Aldrich, ATP synthase inhibitor): 1 µM; FCCP (370-86-5, Sigma Aldrich, uncoupling agent measuring the maximal respiration capacity): 0.5 µM; antimycin A (A8674, Sigma Aldrich, mETC inhibitor): 1 µM.

### Small interfering RNA transfection

HMLER CD24L cells were seeded at a density of 30 000 cells per well in a 12-well plate. After 24 h of pre-incubation cells were transfected at sub-confluence with 10 nM of si-CTRL (D001810-10-20, ThermoFisherScientific, ON-TARGET™*plus* control), si-RAPTOR (sc-44069, Santa Cruz), si-SIN1 (sc-60984, Santa Cruz) were mixed with 3 μL/well of Lipofectamine RNAiMAX (13778-150, Invitrogen) diluted in Opti-MEM (51985-042, Gibco) following the manufacturer’s instructions. After 4 h of transfection, the mix was replaced with fresh medium. The next day a second transfection with the same siRNAs was performed using the same protocol, but after 4 h of transfection, 500 uL of fresh medium was added. Cells were treated the next day as indicated in Fig. 1. Cells were then collected and analyzed by flow cytometry and/or Western blot at corresponding time.

### RNA analysis

Total RNA was extracted from cell pellets using the Nucleospin RNA kit (740955, Macherey-Nagel - Hoerdt) according to the manufacturer’s protocol and quantified using a NanoDrop 2000 spectrophotometer (Thermo Fisher Scientific). cDNA was generated from the total RNA (250 ng) using random hexamer primers (S0142, Thermo Fisher Scientific) and the M-MLV reverse transcriptase (28025-016, Invitrogen) according to the manufacturer’s protocol. mRNA levels of target genes were quantified by qPCR using iTaq Universal SYBR Green Supermix (172-5124, BioRad) according to the manufacturer’s protocol in a CFX96 thermal cycler (BioRad). The data were normalized to the internal control β-actin. Relative gene expression levels were calculated using the 2-ΔΔCt method. Primers used: FTH1 (Forward: 5’-CTGGAGCTCTACGCCTCCTA-3’; Reverse: 5’-TCTCAGCATGTTCCCTCTCC-3’); NCOA4 (Forward: 5’-CAGCTGGTGAGTCGGTGAC-3’; Reverse: 5’-TCCGTGCATCACTACACCTC-3’); TfR-1 (Forward: 5’-GAGCCTGTGGGGAAGGG-3’; Reverse: 5’-AGGCTGAACCGGGTATATGA-3’); β-actin (Forward: 5’-AAGACCTGTACGCCAACACA-3’; Reverse: 5’-TGATCTCCTTCTGCATCCTG-3’).

### Immunoblotting

Cell lysates were prepared on ice in an appropriate lysis buffer (50 mM Tris-HCl, pH 7.5, 150 mM NaCl, 1% TRITON X-100, 0.5% NP-40, 10% glycerol, 1% protease, and a Phosphatase Inhibitor Cocktail (78442, Thermo Fisher Scientific)). Protein concentrations were determined with the Pierce BCA protein assay kit (23225, Thermo Fisher Scientific). Equal protein amounts (15–20 µg) diluted in a 4× Laemmli buffer were denatured by heating at 95 °C for 5 min and separated by electrophoresis on 4–12% NuPAGE Bis-Tris Gel, and then transferred onto a 0.45 μm a PVDF membrane. Membranes were blocked with 5% non-fat dry milk in PBS-T (D-PBS with 0.1% Tween-20) for 1 h at room temperature and then incubated with primary antibodies at 4 °C overnight. Membranes were then washed with DPBS-T, and incubated with the appropriate HRP-coupled secondary antibody for 1 h 30 min at RT. Antibodies are listed in Supplementary Table S3. Membranes were then washed with PBS-T, and bound antibodies were detected using an ECL detection kit (Immobilon Western ECL, Millipore) or with the ChemiDoc Imaging Systems (BioRad) using the CCD camera for light capture according to the manufacturer’s protocol. Signals were quantified using Image Lab Software (Bio-Rad) and normalized to Tubulin or GAPDH.

### Transmission electron microscopy

Cells were treated in a 6-well plate as indicated during 48 h. For ultrastructural analysis, cells were fixed with 1.6% glutaraldehyde in 0.1 mol/L phosphate buffer. After scraping, cell pellets were secondary fixed with 2% osmium tetroxide and dehydrated using ethanol. Cells were embedded in Epon 812 resin. Polymerization was complete after 48 h at 60 °C. Ultrathin sections were collected on 100-mesh grids coated with Formvar and carbon, and stained with standard uranyl acetate and lead citrate solutions. The sections were then examined under a FEI Technai Spirit transmission electron microscope at 80 Kv, and images were acquired with a SIS Mega view III CCD camera.

### Immunofluorescence microscopy

Cells were seeded at a density of 25,000 cells per well on glass coverslips in a 12-well plate, at sub-confluence cells were treated. Then after 48 h, cells were washed twice with PBS and fixed with 4% paraformaldehyde (15714, Electron Microscopy Sciences) for 13 min at 37 °C. After washing twice with PBS, cells were permeabilized with PBS + 10% FBS and 0.3% TRITON X-100 (X100, Sigma Aldrich) at room temperature for 20 min. After washing twice with a washing buffer (PBS + 10% FBS), cells were incubated at 4 °C overnight with the indicated primary antibodies, including anti-TOM20 (1:75), anti-Hsp60 (1:100), or LAMP2 (1:100). Cells were then washed three times and incubated with Alexa 488/647-labeled anti-mouse/rabbit secondary antibody (Invitrogen, A21202, A31573) for 1 h at RT. All antibodies were diluted with a washing buffer. The cells were then washed twice, incubated with DAPI (1:2000) 10mn in PBS, washed twice with PBS and slides were mounted with a coverslip using fluoromount-G medium (0100-20, SouthernBiotech). After adhering, slides were allowed to dry overnight and stored at 4 °C in dark to prevent photobleaching. Cell images were obtained using a Spinning Disk Zeiss or a Confocal Leica SP8 with objective 63X oil. Stacks of images were collected every 0.22 μm (or 0.01 μm for the TOM20) along the z-axis. Images were processed by ImageJ software.

### Mitochondria analysis

Mitochondrial morphology qualification and quantification was performed using two supervised machine-learning (ML) modules to segment mitochondria and classify them on their morphology. Firstly, images were prepared with a FIJI macro. Then, with the “pixel classification + object classification” ML module of Ilastik (V1.3.3post3), on 3D images of z-stack of TOM20, mitochondrial network was segmented into objects. The segmentation was carried out using a supervised ML trained to identify three classes of object: background, mitochondria edges, and mitochondria body, based on several properties/features: Color/Intensity, Edge, Texture. The ML was performed for each experiment on at least 10 cells by condition, before being applied to each experiment independently. Mitochondria were then segmented using a Hysterisis thresholding approach. The mitochondria body recognized as labeled objects were saved as new 3D images. Then, we used Imaris V9.9 Software (Oxford Instruments). Following, mitochondria body were classified into three classes: fragmented, hyperfused, and intermediate, using a supervised ML training based on several shape’s features. This ML was performed on at least 20 cells/condition, before being applied to all the experiments. Finally for each cell, the total volume of mitochondria was compared with the volume of each class. Data were analysed with Graphpad Prism and organised cell the proportional area for each class. Except for the classification of body mitochondria, the analysis of lysosomal area was carried out in the same manner. Then, the masks/area of mitochondria and lysosomes were overlaid by using Fiji image processing software, and the percentage of colocalization per condition and per cell was calculated relative to the total volume of mitochondria.

### Mitochondrial DNA quantification

Cells were treated as indicated during the desired time, then detached with trypsin, collected and rinsed with PBS. The total DNA was extracted by using DNeasy Blood & Tissue Kit (# 69,504, Qiagen, Germany), following manufacturer’s instructions. Quantification of mitochondrial DNA (mtDNA) and nuclear DNA (nDNA) were performed by qPCR with SYBR green-based detection (Thermo Fisher Scientific) using iTaq Universal SYBR Green Supermix (172-5124, BioRad) according to the manufacturer’s protocol in a qTOWER 2.0/2.2 thermal cycler (Analytic Jena). Relative mDNA:nDNA ratio was calculated using the 2^−ΔΔCt^ method upon targeting of nuclear-encoded gene (human B2M) and mitochondrial-encoded gene (human COX1) (Quiros, PM., Goyal A. et al., Curr. Protoc. Mouse Biol.). The sequences of the primers are the following: forward primer: 5’- CCCACCGGCGTCAAAGTAT −3’ and reverse primer: 5’- TGCAGCAGATCATTTCATATTGC −3’ for COX1; and forward primer: 5’- TGCTGTCTCCATGTTTGATGTATCT −3’ and reverse primer: 5’- TCTCTGCTCCCCACCTCTAAGT −3’ for B2M.

### Bioinformatics analysis on proteomic data

Bioinformatics analyses were performed in R software environment version 4.2.1. Based on annotated proteomic matrix and taking in account the experimental groups (Unt.: Untreated; Torin: Tor.; Salinomycin: Sal; Torin + Salinomycin: TorSal) a supervised leave one out process of machine learning was performed by shrunken centroid determination with pamr R-package [58] version 1.56.1. A minimal signature with optimal threshold was determined for a minimal misclassication error of the samples between supervised experimental groups. A quick decrease of misclassification error was observed in four experimental groups (Supplementary Fig. S4A). A minimal proteomic signature with threshold fixed to 6 of value (Supplementary Fig. S4A) was retained. Minimal signature was validated by unsupervised principal component analysis performed with « prcomp » r-base function and plotted with « autoplot » function from ggfortify R-package version 0.4.14. This minimal proteomic signature allowed us to obtain a perfect classification of the groups after cross-validation by leave one out process (Supplementary Fig. S4B). Based on this signature, a good stratification of the samples between groups according to their cross-validated probabilities was performed (Supplementary Fig. S4C). The minimal signature was also validated by unsupervised clustering (Pearson distances) performed with heatmap R-package version 1.0.12. Network functional enrichment was done with Kyoto Encyclopedia of Genes and Genomes (KEGG) database [59].

### Quantification and statistical analysis

All results were expressed as mean values ± SD and were compared by one-way or two-way ANOVA or an unpaired two-tailed Student’s *t*-test. Analyses were performed using Graph Pad Prism 9.0. Differences were considered to be statistically significant when *P* < 0.05.

### Supplementary information

Legends of Supplementary Figures and Tables

Supplementary Figures

Supplementary Table S1

Supplementary Table S2

Supplementary Table S3

Original Data File

aj-checklist

## Data Availability

All data supporting the findings of this study are available within the paper and are available from the corresponding author upon request. Any additional information required to reanalyze the data reported in this paper is available from the lead contact upon request.

## Material availability

All request for resources and reagents should be directed to and will be fulfilled by the lead contact. All reagents will be made available on request after completion of a Materiel Transfer Agreement.
