# Supplementary material for: mTOR inhibition suppresses salinomycin-induced ferroptosis in breast cancer stem cells by ironing out mitochondrial dysfunctions
Source: Cell Death Dis. 2023 Nov 15;14(11):744. doi: 10.1038/s41419-023-06262-5 (PMC10651934; doi:10.1038/s41419-023-06262-5)
Supplement: Supplementary file 1 — Legends of Supplementary Figures and Tables [file 41419_2023_6262_MOESM1_ESM.docx]

**Legends of Supplementary Figures and Tables**

**Supplementary Fig. S1., related to Figure 1. mTOR inhibition inhibits ferroptotic cell death induced by Sal. (A)** HMLER CD24L cells were treated with Sal (500 nM), Torin (250 nM) or combination of both. Cell death was determined by Annexin-V and Propidium Iodide (PI) staining coupled with flow cytometry (FC) after 96h (n=3). Live cells were identified as (Annexin V^-^ / PI^-^) cells; necrotic cells as (Annexin V^-^ / PI^+^) cells; early apoptotic cells as (Annexin V^+^ / PI^-^) cells; and necrotic/late apoptotic cells as (Annexin V^+^ / PI^+^) cells. **(B)** HMLER CD24L were treated with either Sal, Torin or combination of both for 48h. Protein level detected by immunoblot and densitometry analysis normalized on Tubulin level. **(C)** HMLER CD24L were pretreated or not with either antioxidant ferrostatin-1 (Fer-1; 2 μM), liproxstatin-1 (Lip-1; 2 μM), or vitamin E (VitE; 125 μM) for 4h, and Sal was added or not for another 78h. Cell viability was analyzed as described in (A). Cell viability represented the percentage of live cells (Annexin V^-^ / PI^-^) (n=4). **(D)** HMLER CD24L were treated with either Sal, Torin or combination of both for 48h. PTGS2/COX2 Protein levels are detected by immunoblot and Actin level is used as loading control. **(E-F)** HMLER CD24L were treated for 96h with Sal with or without **(E)** Torin 250nM or Rapamycin 250nM **(F)** left panel, Torin-2 (250 nM); right panel, AZD8055 (250 nM). Cell death determined by dapi staining coupled with flow cytometry (FC). **(G)** HMLER CD24L were treated with either Sal, Torin (250 nM), Torin-2 (250 nM), AZD8055 (250 nM) or combination of Sal with each mTor inhibitor for 48h. Immunoblot of the mTOR-dependent phosphorylation of ribosomal protein S6 supporting the action of mTOR inhibitors. GAPDH level is used as a loading control.

**Supplementary Fig. S2., related to Figure 2. mTOR inhibition impacts iron homeostasis dysregulation induced by Sal.** **(A-D)** HMLER CD24L were treated with either Sal, Torin or combination of both for 48h. **(A)** Lipid ROS level determined by BodipyC11 staining coupled with FC (n=5). **(B-C)** HMLER CD24L knockdown for either *RAPTOR* or *SIN1* and then treated as indicated. **(B)** Global ROS levels determined by H2-DCFDA staining coupled with FC (n=5). **(C)** Fe^2+^ levels determined by FerroOrange staining coupled with FC (n=5). **(D)** HMLER CD24L were pretreated or not with either iron chelator IV (1 and 2 μM), or deferoxamine (DFO; 10 μM) for 4h, and Sal was added or not for another 78h. Cell death was determined by dapi staining coupled with flow cytometry. **(E)** HMLER CD24L were treated with Sal, Torin or combination of both with or without FeCl3 (500 μM) for 96h. Cell death was determined by dapi staining coupled with flow cytometry (FC) (n=4). **(F)** Protein level of TfR detected by immunoblot and densitometry analysis normalized on Tubulin level. **(G)** Gene expression level of TFRC detected by RT-qPCR normalized on Actin level. **(H)** Protein level of FTH detected by immunoblot and densitometry analysis normalized on Tubulin level. **(I)** HMLER CD24L were treated with either Sal or/and Torin in combination with HCQ (50 μM or 100 μM) for 48h. Immunoblotting for the indicated autophagy-related protein. Tubulin was used as a loading control. **(J)** HMLER CD24L were treated with either Sal or/and Torin in combination with HCQ (50 μM) for 96h. Cell death was determined by dapi staining coupled with flow cytometry (FC). Data are presented as: mean ±SD, ANOVA test: *p < 0.05; **p < 0.01; ***p < 0.001; ****p < 0.0001.

**Supplementary Fig. S3., related to Figure 3. Enrichment map of GO terms enriched in proteins downregulated by Sal treatment (and restored by Sal + Tor combining treatment) from Cluster A shown in an interaction network.** MF, Molecular Function; CC, Cellular Component; and BP, Biological Process.

**Supplementary Fig. S4., related to Figure 3. A regulation of 187 proteins discriminates the four experimental conditions investigated by proteomic analysis.** **(A)** Supervised machine learning miss-classification error by class according number of proteins included in the model. **(B)** Confusion matrix obtained with the 187-protein signature for optimal threshold of 6. (**C**) Plot cross validated probabilities for samples obtained with the 187-protein signature. **(D)** Bar plot of normalized False Discovery Rates obtained on 187 protein-network enriched with KEGG database. **(E)** Bar plot of molecule counts obtained on 187 protein-network enriched with KEGG database.

**Supplementary Fig. S5., related to Figure 3. Sal treatment dramatically affects mitochondrial protein expression which is completely rescued by mTOR inhibition. (A)** Heatmap comparing relative levels of 321 mitochondrial proteins from 1919 proteins significantly modulated by Sal treatment, Tor. alone treatment, and combination treatment compared to untreated (Unt.) cells for 48 h. A focus of some mitochondrial proteins modulated by Sal treatment is shown: red, upregulated; green, downregulated; black, no modulated as compared to untreated cells. **(B)** Heatmap comparing relative levels of proteins from 321 mitochondrial proteins significantly modulated by Sal treatment, Tor. alone treatment, and combination treatment compared to untreated (Unt.) cells for 48 h. These proteins were clustered in function of their role or their category in mitochondria as indicated. Color key indicates protein expression value (green: lowest; red: highest). Proteins were clustered using Perseus software.

**Supplementary Fig. S6, related to Figure 5. mTOR inhibition protects from mitochondrial damages induced by Sal.** **(A-F)** HMLER CD24L were treated with either Sal, Torin or combination of both for 48h. **(A)** Immunoblotting for the indicated ETC-related protein. GAPDH was used as a loading control and **(B)** quantification by densitometry analysis normalized on GAPDH level. **(C)** Quantification of mitochondrial morphology. Analysis on >25 cells from 2 independent experiments. **(D)** The mitochondrial mass was measured by mitoDNA (mDNA, MT-COX1) content. mtDNA was examined by qPCR and normalized by nuclear DNA (nDNA, B2M). Shown are mean ± SEM (n=3 biological replicates). Two tailed and unpaired student’s t test. *, P<0.05, **p < 0.01; ns, not significant. (**E**) TEM images (scale bars: 1μm, 500 nm). Red stars indicate autophagic vacuoles/autolysosomes. m, mitochondria. (**F**) Upper panel, immuno-staining of mitochondria with Hsp60 and lysosome with Lamp2; images acquired using Spinning Disk Zeiss. Nuclei were counterstained with Dapi. Objective: 63x. Scale bar: 20 μm and 5 μm. Lower panel, graphs representing lysosomal area (left), mitochondrial area (middle), and the percentage of colocalization between lysosomes and mitochondria (reported as number of colocalized pixels per mitochondrial area) (n=16 cells each condition). ANOVA test: *p < 0.05; **p < 0.01; ***p < 0.001; ****p < 0.0001. (**G**) HMLER CD24L were treated with either Sal or/and Torin in combination with HCQ (50 μM or 100 μM) for 48h. Immunoblotting for NDP52, BNIP3, OPTN, and TIM23. Tubulin was used as a loading control.

**Supplementary Tables**

**Supplementary Table S1: List of 1919 proteins differentially expressed between the four experimental conditions with a 2-fold change.**

**Supplementary Table S2: List of the 187 proteins from the discriminant signature between the four experimental conditions of proteomics.**

**Supplementary Table S3: List of Reagents and Antibodies.**
