# Supplementary material for: mTOR inhibition suppresses salinomycin-induced ferroptosis in breast cancer stem cells by ironing out mitochondrial dysfunctions
Source: Cell Death Dis. 2023 Nov 15;14(11):744. doi: 10.1038/s41419-023-06262-5 (PMC10651934; doi:10.1038/s41419-023-06262-5)

Figure S1 - related to Fig 1

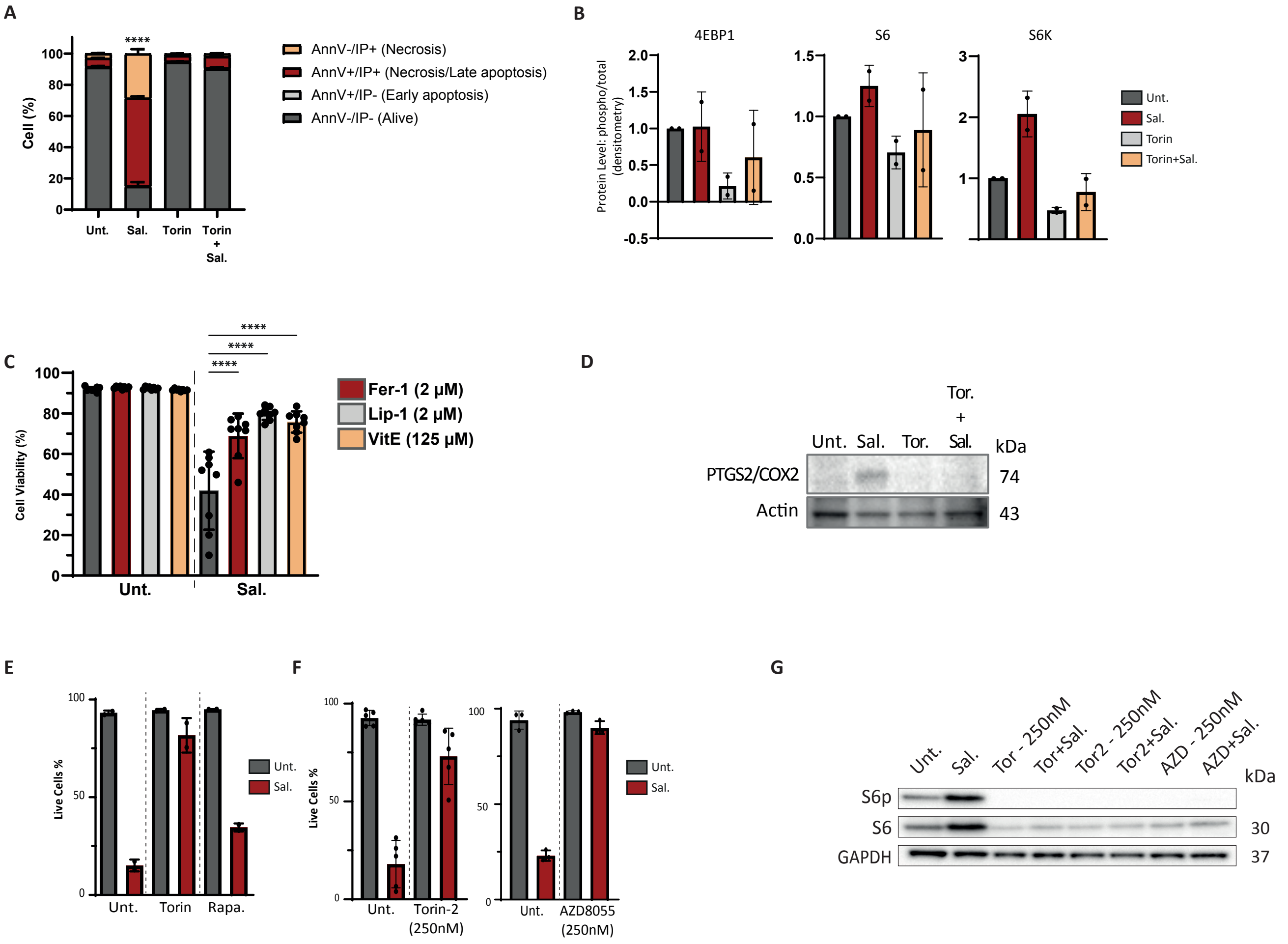

Figure S2 - related to Fig 2

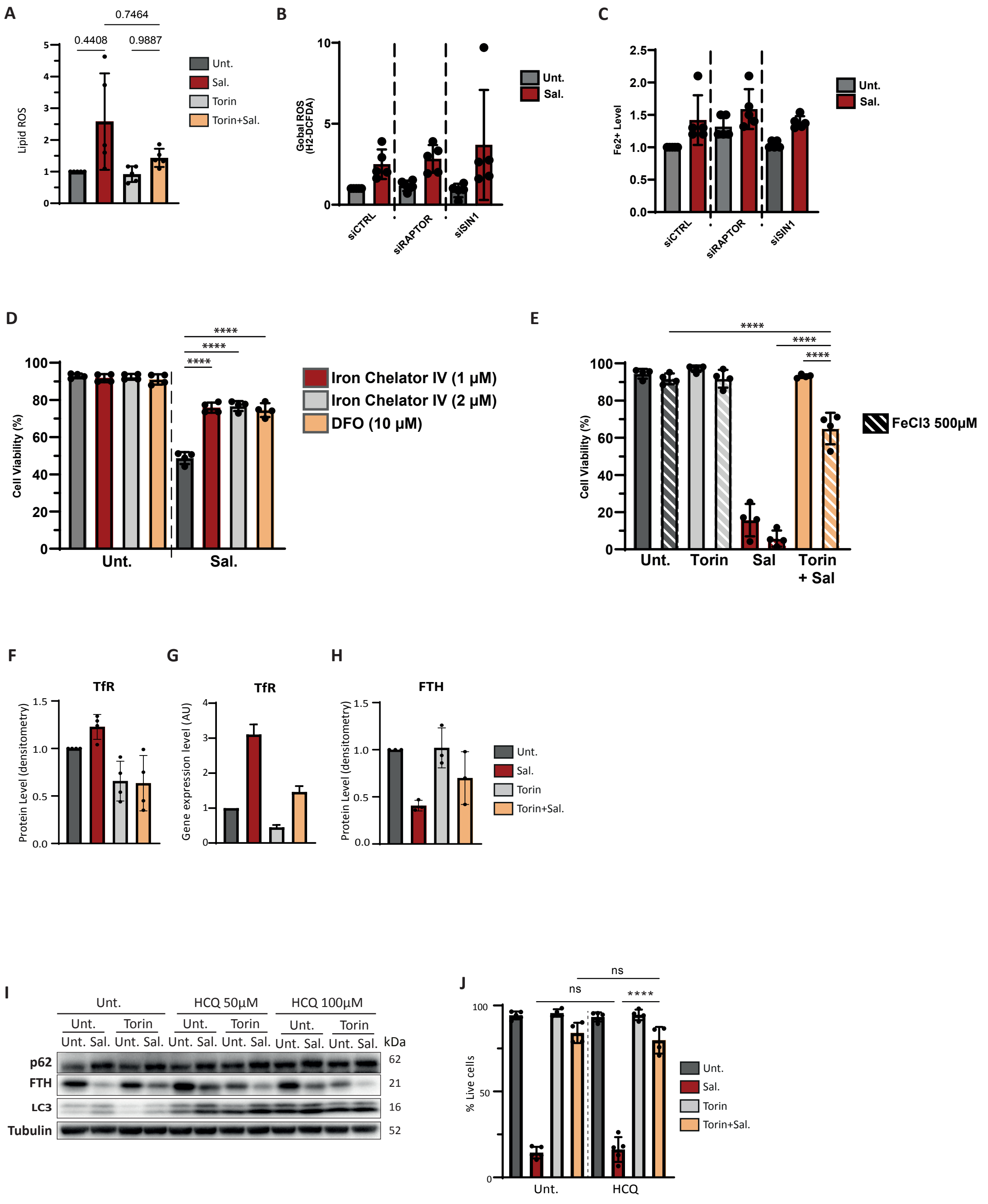

Figure S3 - related to Fig 3

GO: MF enrichment

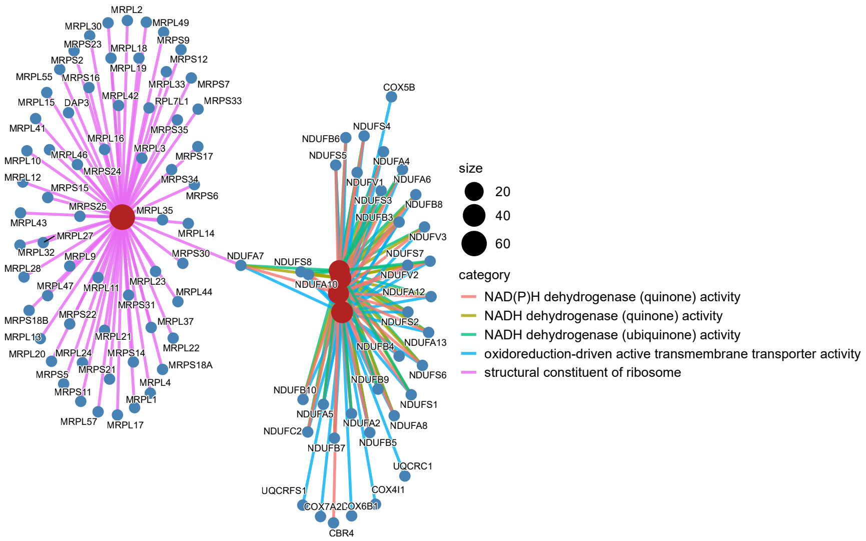

GO: CC enrichment

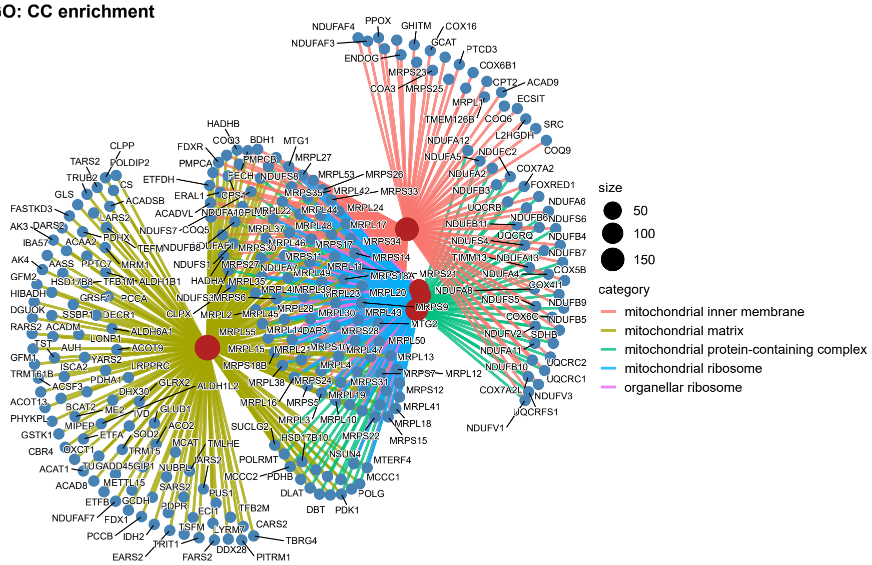

GO: BP enrichment

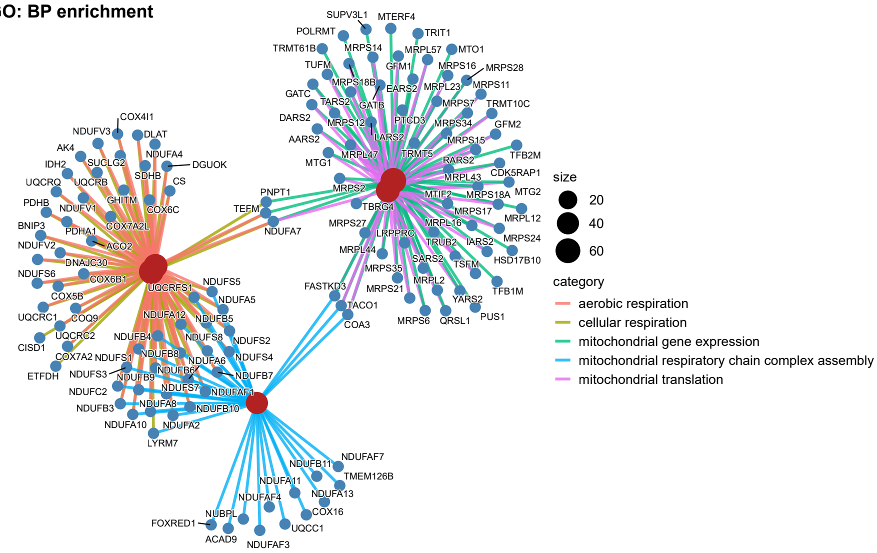

Figure S4 - related to Fig 3

A

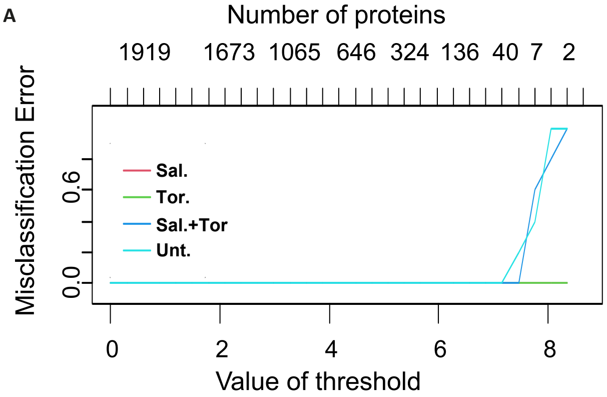

B

misclass error = 0	Sal.	Tor.	Sal.+Tor	Unt.	class error
Sal.	**5**	0	0	0	0
Tor.	0	**5**	0	0	0
Sal.+Tor	0	0	**5**	0	0
Unt.	0	0	0	**5**	0

C

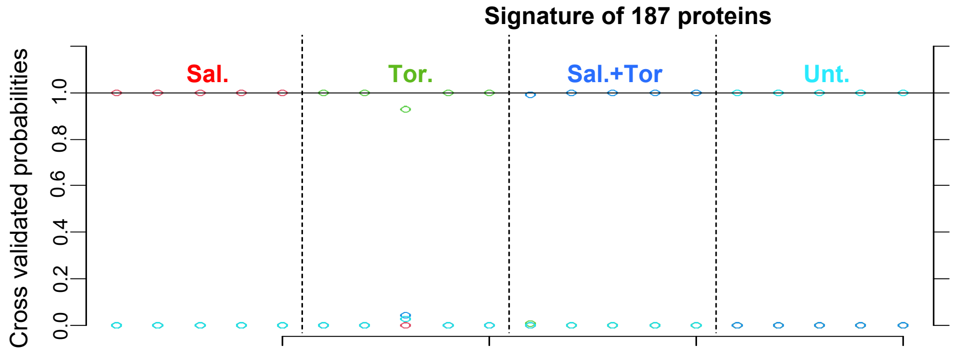

D

E

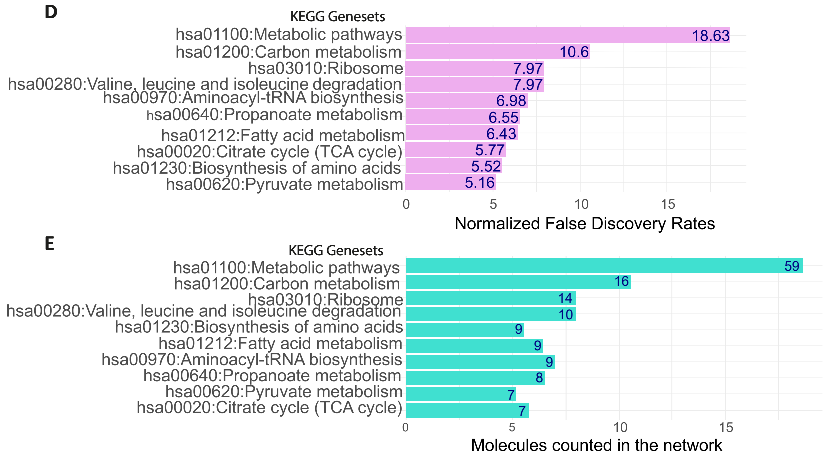

Figure S5 - related to Fig 3

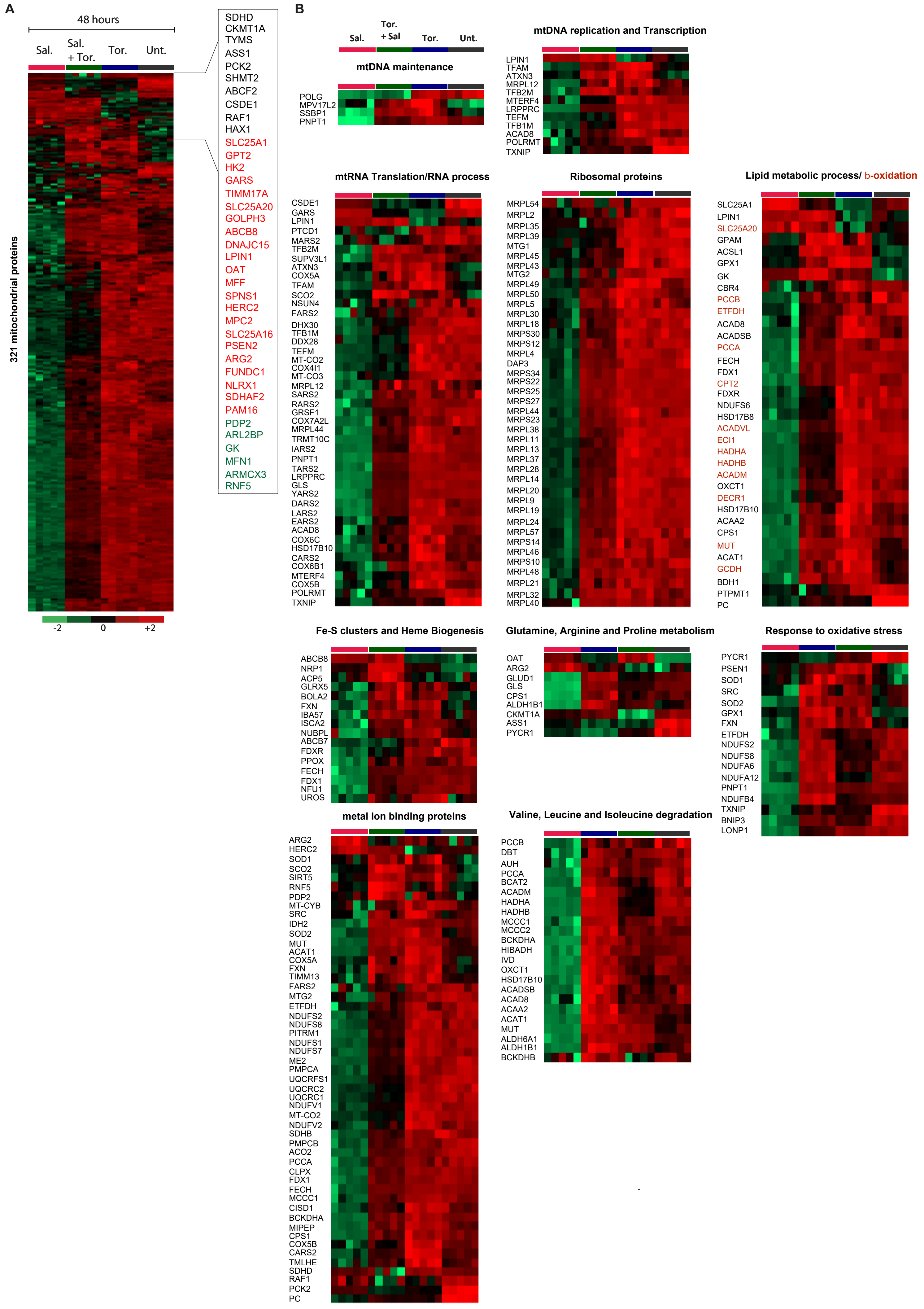

Figure S6 - related to Fig 5

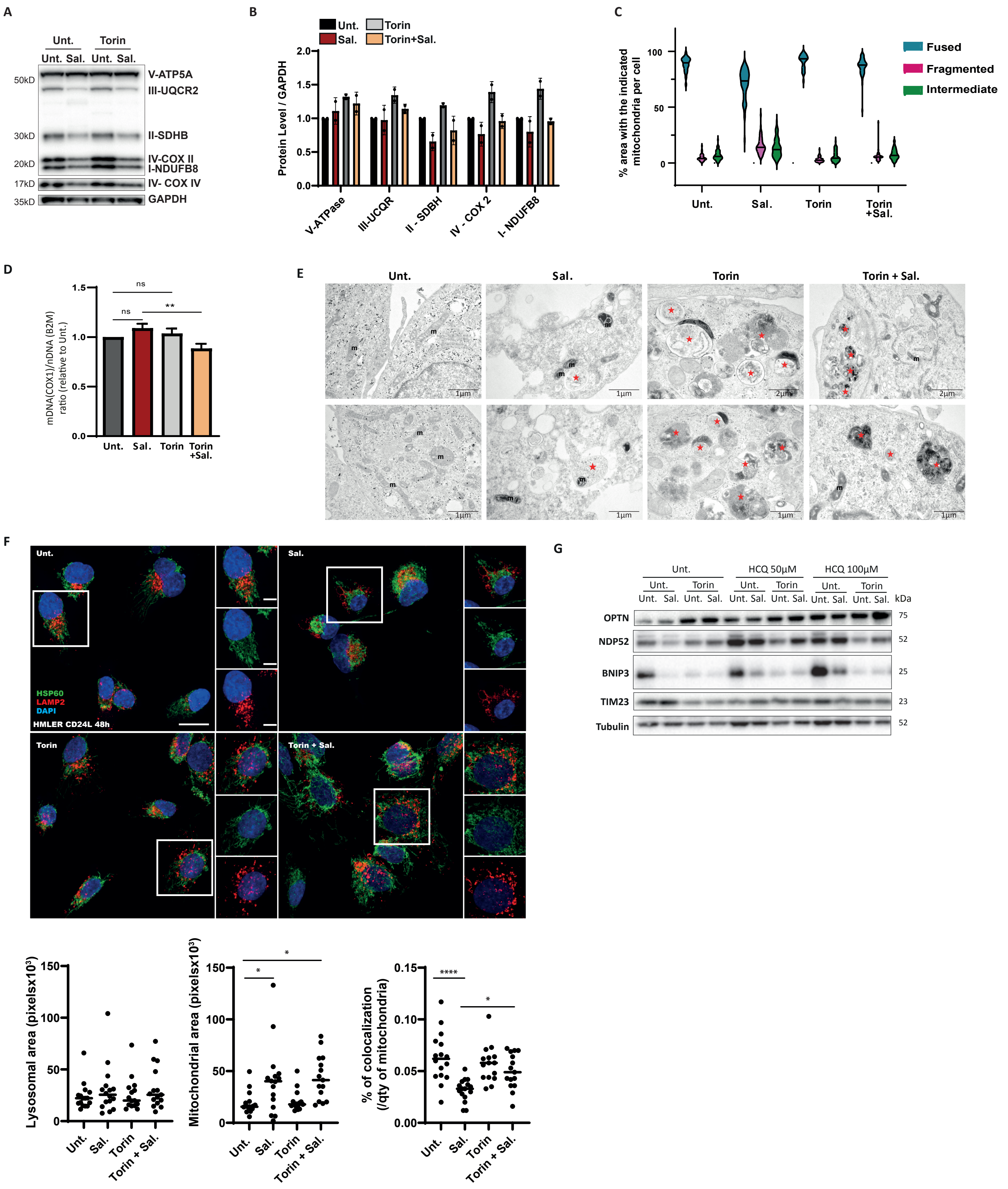

Supplement: Supplementary file 2 — Supplementary Figures [file 41419_2023_6262_MOESM2_ESM.pdf]
