# Supplementary material for: mTOR inhibition suppresses salinomycin-induced ferroptosis in breast cancer stem cells by ironing out mitochondrial dysfunctions
Source: Cell Death Dis. 2023 Nov 15;14(11):744. doi: 10.1038/s41419-023-06262-5 (PMC10651934; doi:10.1038/s41419-023-06262-5)
Supplement: Supplementary file 5 — Supplementary Table S3 [file 41419_2023_6262_MOESM5_ESM.pdf]

**Supplementary TABLE S3**

| |
|---|
| 4EBP1 (Cell Signalling #9644, WB :1/1000) |
| 4EBP1 - phospho (Thr37/46) (Cell Signalling #2855, WB : 1/1000 in 5% BSA) |
| ATG3 (SAB3500500, Sigma Aldrich, WB :1/1000) |
| ATG7 (Sigma Aldrich A2856, WB: 1:1000) |
| ATG9A (PD042, MBL International, WB : 1/1000) |
| BNIP3 (Santa Cruz, sc-56167, WB : 1/1000) |
| BNIP3L (12393, Cell Signaling, WB : 1 :500) |
| DRP1 (BD Biosciences B611113, WB : 1/1000) |
| FTH (Abcam 75973, WB : 1/1000) |
| GAPDH (Santa Cruz sc-47724, WB : 1:2000) |
| Hsp60 (12165, Cell Signaling, WB : 1:500, IF : 1/100) |
| IRP2 (Cell Signalling #37135, WB : 1/1000 in 5% BSA) |
| LAMP2 (Developmental Studies Hybridoma Bank, AB_528129, IF: 1/100) |
| LC3 (Sigma L7543, WB : 1/1000) |
| MFN2 (Cell Signaling, 9482, WB : 1/1000) |
| NCOA4 (Santa Cruz sc-373739, WB : 1/1000) |
| NDP52 (Proteintech, 12229-1-AP, WB : 1/1000) |
| OPA1 (Cell Signalling, #80471, WB : 1/1000) |
| OPTN (Proteintech 10837-1-AP, WB : 1/1000) |
| p62 (Progen - GP62-C, WB : 1/1000) |
| p70-S6K (Cell signaling #9202, WB : 1/1000) |
| p70-S6K-phospho (Thr389) (Cell Signalling #9205, WB : 1:500 in 5% BSA) |
| phospho-S6 (Ser240/244) (Cell signalling #5364, WB : 1/1000 in 5% BSA) |
| PTGS2/COX2 (Cell signaling, 12282, WB: 1/1000) |
| R-TF1/TFRC (H68.4) (ThermoFisher #13-6800, WB : 1/1000) |
| Raptor (Invitrogen #42-4000, WB : 1/1000) |
| S6 (Cell signalling #2217, WB : 1/1000) |
| Sin1 (Cell signalling #12860, WB : 1/1000) |
| TIM23 (611223, BD Transduction Laboratories, WB : 1/1000) |
| TOM20 (BD Biosciences 612278, WB : 1/1000, IF : 1/75) |
| TOMM40 (18409-1-AP, Proteintech, WB : 1/1000) |
| Total OXPHOS Cocktail (Abcam 110411, WB : 1/1500) |
| Tubuline (Sigma T5168, WB : 1/2000) |
| VHL (BD Biosciences 556347, WB : 1/1000) |
| **Secondary Antibodies (Source, Reference)** |
| Anti-Rabbit IgG, HRP-linked (Cell Signaling #7074, WB: 1:2000) |
| Anti-Mouse IgG, HRP-linked (Cell Signaling #7076, WB: 1:2000) |
| **Drugs (Source, Reference)** |
| AZD-8055 (MedChamExpress, #HY-10422) |
| Compound 968 (Merck Millipore, 352010) |

| |
|---|
| Deferoxamine (DFO, Sigma-Aldrich, D9533) |
| Ferrostatin-1 (Fer-1, Sigma-Aldrich, SML0583-5MG) |
| Liproxstatin-1 (Lip-1, Sigma-Aldrich, SML1414-5MG) |
| MHY 1485 (Sigma-Aldrich, SML0810) |
| Rapamycin (Rapa., Sigma-Aldrich, R0395) |
| Salinomycin (Sal, Sigma-Aldrich, S46729) |
| Torin-1 (Torin, Tocris Bioscience, 4247) |
| Vitamin E/α-Tocopherol (VitE., Sigma-Aldrich, 258024) |
| **Chemical Reagents (Source, Reference)** |
| CM-H2DCF-DA (20,7'-dichlorodihydrofluorescein diacetate, Invitrogen, C6827) |
| HPF (Hydroxyl Radical and Peroxynitrite Sensor, Thermofisher H36004) |
| Bodipy 581/591 C11 (Invitrogen, D3861) |
| DAPI (4',6-diamidino-2-phenylindole, Invitrogen, D1306) |
| FeCl3 (Sigma-Aldrich, 451649) |
| FerroOrange (Dojindo, F374) |
| Mito-Ferro (Dojindo, M489) |
| Alexa-488-Transferin (Tf, ThermoFisher Scientific, T13342) |
| MitoC11 (MitoPerox, Abcam, ab146820) |
| Mitotracker Green FM (Thermofisher, M7514) |
| Mitotracker CMXRos (Thermofisher, M7512) |
| NucLight Rapid Red probe (Essen BioScience, 4717) |
| Cytotox Green probe (Essen BioScience, 4633) |
| **Experimental models (Source, Reference)** |
| HMLER CD24Low (gift from A. Puisieux, [55]) |
| **Software and algorithms** |
| GrapghPad Prism 9.0 (GraphPad Sotware, Inc, https://graphpad.com/scientific-software/prism/ |
| Excel 2020 (Microsoft, https://www.office.com/) |
| ImageJ 1.52a (Wayne Rasband NIH, https://imagej.net) |
| BD FACS DIVA 6.2 (BD Biosciences, http://www.bdbiosciences.com/en-us/intruments/research-instruments) |
| FlowJo (BD Biosciences, http://www.flowjo.com) |
