# Supplementary material for: mTOR inhibition suppresses salinomycin-induced ferroptosis in breast cancer stem cells by ironing out mitochondrial dysfunctions
Source: Cell Death Dis. 2023 Nov 15;14(11):744. doi: 10.1038/s41419-023-06262-5 (PMC10651934; doi:10.1038/s41419-023-06262-5)

## Full and uncropped western blots for Fig. 1C

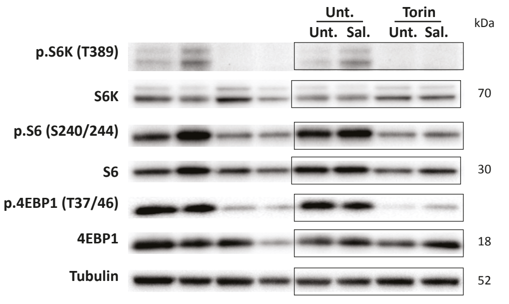


## Full and uncropped western blots for Fig. 1D

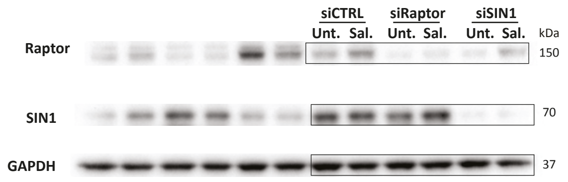


## Full and uncropped western blots for Fig. S1D

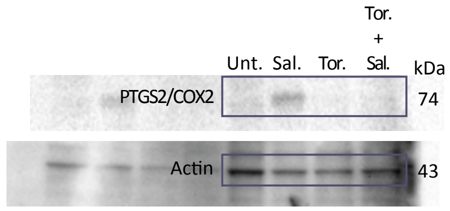


## Full and uncropped western blots for Fig. S1G

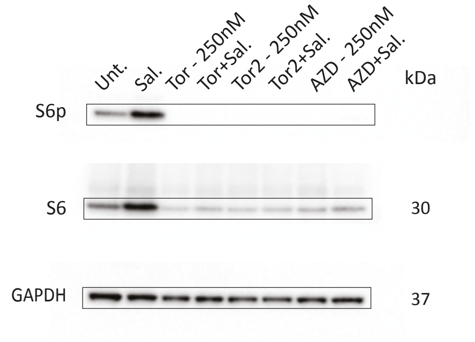


## Full and uncropped western blots for Fig. 2G

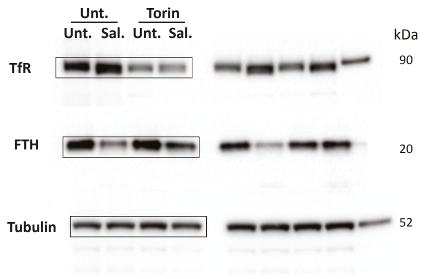


## Full and uncropped western blots for Fig. S2I

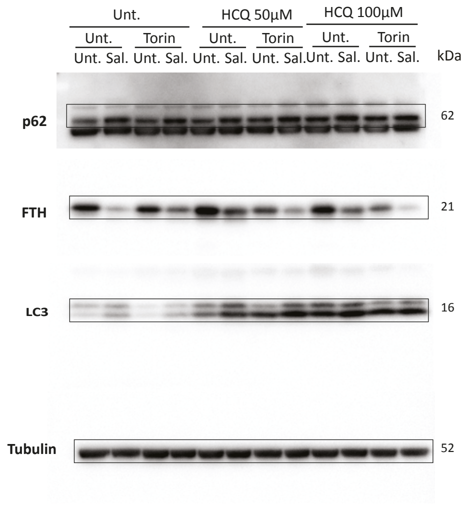

## Full and uncropped western blots for Fig. 5G

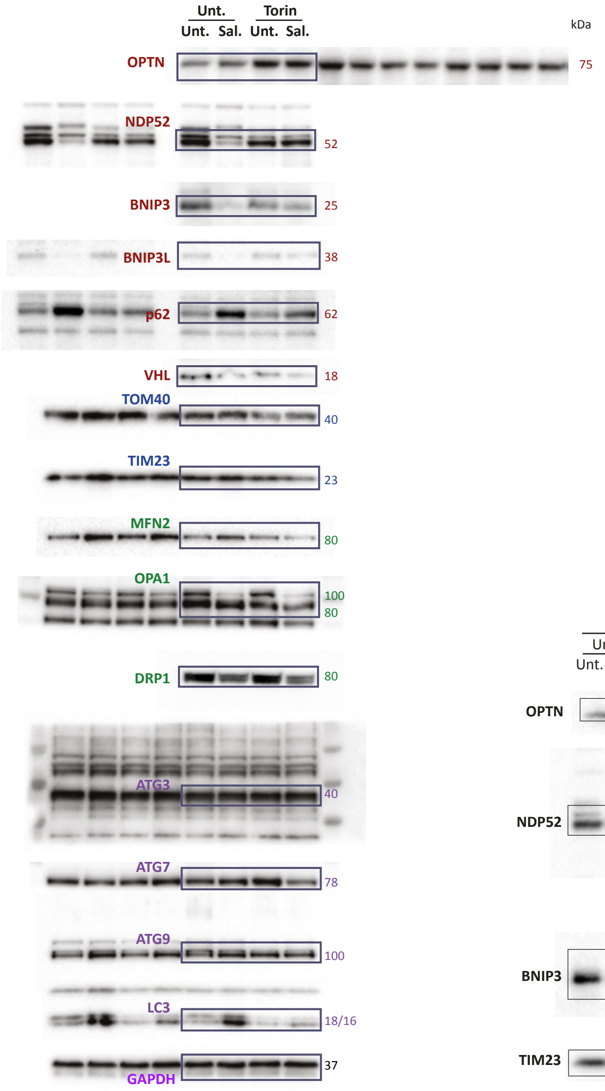


## Full and uncropped western blots for Fig. S6A

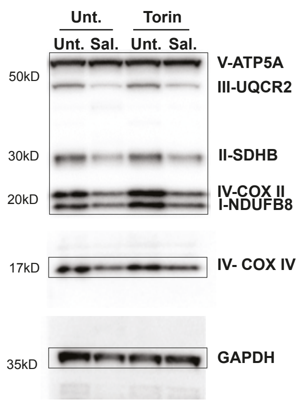


## Full and uncropped western blots for Fig. S6G

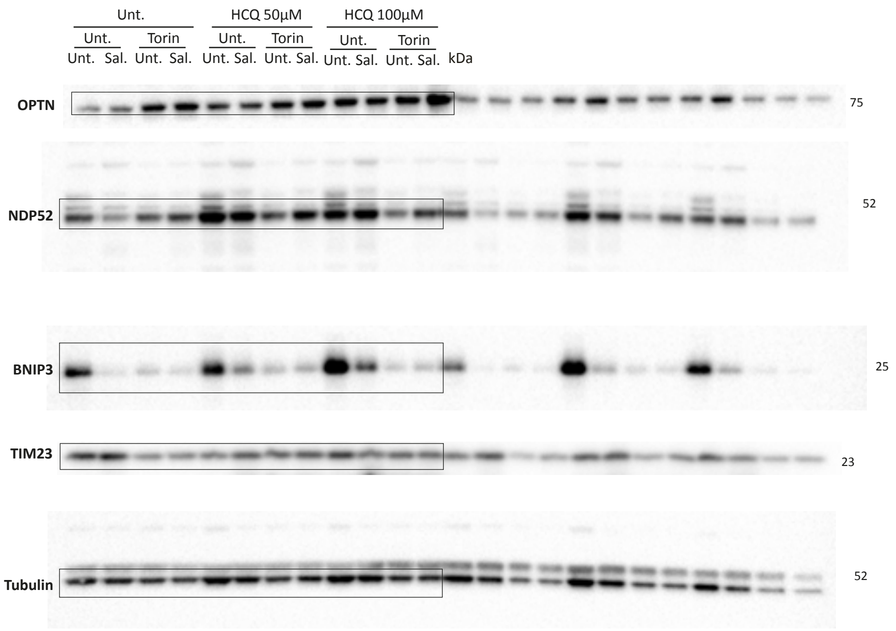

Supplement: Supplementary file 6 — Original Data File [file 41419_2023_6262_MOESM6_ESM.pdf]
